# Materials Engineering of Violin Soundboards by Stradivari and Guarneri

**DOI:** 10.1002/anie.202105252

**Published:** 2021-06-27

**Authors:** Cheng‐Kuan Su, Szu‐Yu Chen, Jen‐Hsuan Chung, Guo‐Chian Li, Brigitte Brandmair, Thomas Huthwelker, John L. Fulton, Camelia N. Borca, Shing‐Jong Huang, Joseph Nagyvary, Hsiao‐Han Tseng, Chih‐Hui Chang, Dai‐Ting Chung, Rafael Vescovi, Yi‐Shiuan Tsai, Wenjie Cai, Bing‐Jyun Lu, Jia‐Wei Xu, Chia‐Shuo Hsu, Jun‐Jie Wu, Hao‐Zhi Li, Yu‐Kai Jheng, Sheng‐Fong Lo, Hao Ming Chen, Yi‐Ting Hsieh, Po‐Wen Chung, Chien‐Sheng Chen, Yuh‐Chang Sun, Jerry Chun Chung Chan, Hwan‐Ching Tai

**Affiliations:** ^1^ Department of Chemistry National Chung Hsing University Taichung Taiwan; ^2^ Department of Optics and Photonics National Central University Taoyuan Taiwan; ^3^ Department of Chemistry National Taiwan University 1 Roosevelt Road Section 4 Taipei 106 Taiwan; ^4^ Hilgertshausen-Tandern Germany; ^5^ Swiss Light Source Paul Scherrer Institut Villigen Switzerland; ^6^ Physical Sciences Division Pacific Northwest National Laboratory Richland USA; ^7^ Instrumentation Center National Taiwan University Taipei Taiwan; ^8^ Department of Biochemistry and Biophysics Texas A&M University College Station USA; ^9^ Chimei Museum Tainan Taiwan; ^10^ Argonne National Laboratory Lemont IL USA; ^11^ Institute of Chemistry Academia Sinica Taipei Taiwan; ^12^ School of Cultural Industry and Tourism Xiamen University of Technology Xiamen Fujian China; ^13^ Department of Chemistry Soochow University Taipei Taiwan; ^14^ Department of Chemistry Fu-Jen Catholic University New Taipei City Taiwan; ^15^ Department of Forestry and Natural Resources National Ilan University I-Lan Taiwan; ^16^ Department of Biomedical Engineering and Environmental Sciences National Tsing-Hua University Hsinchu Taiwan

**Keywords:** cultural heritage, ICP-MS, IR spectroscopy, NMR spectroscopy, X-ray absorption spectroscopy

## Abstract

We investigated the material properties of Cremonese soundboards using a wide range of spectroscopic, microscopic, and chemical techniques. We found similar types of spruce in Cremonese soundboards as in modern instruments, but Cremonese spruces exhibit unnatural elemental compositions and oxidation patterns that suggest artificial manipulation. Combining analytical data and historical information, we may deduce the minerals being added and their potential functions—borax and metal sulfates for fungal suppression, table salt for moisture control, alum for molecular crosslinking, and potash or quicklime for alkaline treatment. The overall purpose may have been wood preservation or acoustic tuning. Hemicellulose fragmentation and altered cellulose nanostructures are observed in heavily treated Stradivari specimens, which show diminished second‐harmonic generation signals. Guarneri's practice of crosslinking wood fibers via aluminum coordination may also affect mechanical and acoustic properties. Our data suggest that old masters undertook materials engineering experiments to produce soundboards with unique properties.

## Graphical abstract

ICP‐MS is used to identify the unique mineral recipe used by Antonio Stradivari to treat his spruce wood, the material for the violin top plate. Chemical manipulation led to hemicellulose fragmentation and cellulose rearrangement. Incorporating engineered wood may contribute to the unique tonal qualities of these acclaimed instruments.

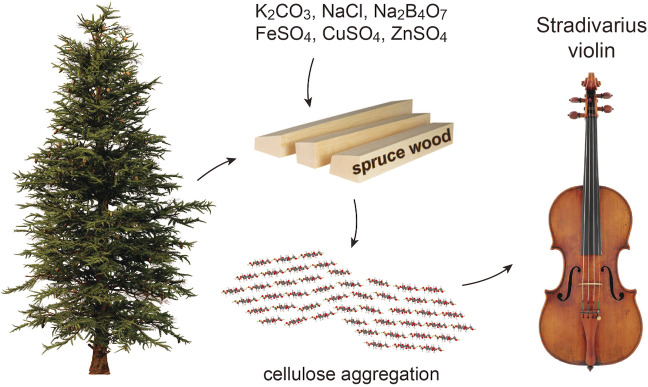

## Introduction

In string instruments, specially selected woods (i.e. tonewoods) act as transducers of mechanical energy from vibrating strings into acoustic energy.^[1]^ Violin‐family instruments, including violas and cellos, are made of two types of tonewoods—Norway spruce (*Picea abies*, for soundboards) and maple (*Acer* species, for ribs and back plates).^[2]^ Curiously, leading violinists today still prefer antique instruments made by two old masters from Cremona, Italy—Antonio Stradivari (1644–1737, Latinized as Stradivarius) and Giuseppe Guarneri “del Gesù” (1698–1744).[^2b^, ^3^] Stradivari also made violas and cellos that are highly prized. Despite tremendous advances in sciences and arts since the industrial revolution, violin making represents a singular case that has undergone a functional decline.^[4]^

After two centuries of investigations, there is still little consensus on what makes Cremonese violins so unique.[^2b^, ^3^, ^5^] Subjective listening tests comparing Stradivari violins to modern instruments have shown mixed results. Blind tests using recorded passages, allowing listeners to do unrestricted A/B switching, have put Stradivari violins in favor,^[6]^ while blind tests with live performances have not.[^6^, ^7^] We have questioned the validity of live listening tests because they fail to address memory decay and loudness matching issues.^[8]^ Our objective measurements have shown that Stradivari violins imitate the vocal tract resonance frequencies of female singers, which may contribute to their perceived brilliance.[^8b^, ^9^] Many speculated that Cremonese masters had developed closely guarded techniques that became lost after 1750.[^2b^, ^5^, ^10^] Investigations into Stradivari violins have mostly focused on the geometry[^2b^, ^5^, ^11^] and varnish compositions,^[12]^ but copying these attributes seems insufficient to reproduce their unique playing qualities.

The soundboard is the most important acoustic component of the violin.[^11b^, ^13^] It also has to withstand over 80 N (approx. 8 kilogram‐force) of string tension exerted downward through the bridge feet.^[14]^ Modern makers usually copy the shapes of Stradivari and Guarneri soundboards but not their thickness. Many Stradivari and Guarneri soundboards appear surprisingly thin and light by modern standards.[^5^, ^15^] The average modern soundboard, made of unaltered, air‐dried spruce,[^5^, ^16^] retains approximately 3.0 mm center thickness to avoid cracking risks over time,[^15d^, ^17^] or even up to 3.5 mm in German schools.^[18]^ In contrast, Stradivari's center thickness range is 2.0–2.8 mm^[5]^ and Guarneri's is 2.2–2.9 mm.^[15a]^ Although some of the Cremonese soundboards could have been scraped thinner during later modifications,[^5^, ^15a^, ^19^] the combination of thinness and durability nevertheless implies distinctive material properties. Such distinctions can neither be attributed to botanical origins nor tonewood selection criteria. Spruce and maple trees are still widely grown in Europe, allowing easy access to high‐quality tonewoods.[^2^, ^5^] Computed tomography (CT) measurements also confirmed that spruce/maple densities are similar between Cremonese and modern instruments.^[20]^ Therefore, we hypothesized that the distinctive properties of Cremonese spruces originated from artificial manipulation.

Recent studies found evidence of unusual elemental profiles and organic composition changes in the maples of Stradivari and Guarneri.^[21]^ However, what chemicals were used to treat the maple and who conducted the treatments remained unclear. This motivated us to analyze a rare collection of Cremonese wood samples that included both spruce and maple from the three major Cremonese families: Amati, Stradivari, and Guarneri (Supporting Information Figure S1). They were compared against modern spruce and maple tonewoods from violin shops as well as aged controls from unexceptional old European violins (18–19th century), old European buildings (18th century), and antique Chinese zithers (ca. 9–10th century; Table 1 and Supporting Information Table S1–S3). We rediscovered the forgotten materials engineering paradigm of Cremonese makers, which involved applying chemical treatments to produce spruce soundboards with unique chemical and physical properties.

**Table 1:** Summary of chemical alterations in Cremonese wood samples.^[a]^

Cremonese instruments	Sample	Al	B	Ca	Cl	Cu	Fe	K	Na	S	Zn	Cellulose rearrangement
Amati viola ca. 1619	spruce SC1		+		++	+	+					No*
Amati viola ca. 1619	maple MC1		+		++		+				+	nt
Strad cello ca. 1701	spruce SC2						+					nt
Strad cello ca. 1707	maple MC2	nt	nt	nt	nt	nt	nt	nt	nt	nt	nt	No^#^
Strad violin ca. 1709	spruce SC3						+			++		nt
Strad violin ca. 1717	maple MC3	+	+		+++	+	+	+++	+++		+	Yes^#^
Strad cello ca. 1720	spruce SC4	+	+		+++	+	+	+++	+++		+	Yes*
Strad violin ca. 1725	maple MC4				+++		ic	+++	+++	++	+	Yes^#^
Strad violin ca. 1730	spruce SC5						+					nt
Strad cello ca. 1731	maple MC5		+		++							No^#^
Guarneri violin ca. 1740	spruce SC6	+++		+++			++			++		nt
Guarneri violin ca. 1741	maple MC6	+++		++	nt	+	+			nt	+	nt

Control samples (see Tables S1 and S2 for details)												
Modern spruce tonewood						SM1–SM5						
Aged spruce from antique instruments						SA1–SA4						
Aged spruce from old buildings						SO1–SO3						
Modern maple tonewood						MM1–MM7						
Aged maple from antique instruments						MA1–MA5						

[a] Blank indicates no apparent change compared to modern and age‐matched controls. Increases in elemental concentrations are approximately divided into three ranges: +(tens of ppm); ++(hundreds of ppm); and +++(thousands of ppm). Here, “ic” indicates possible iron contamination due to the original nails; “nt” indicates not tested due to limited sample amounts. Cellulose rearrangement was detected by SHG spectroscopy in this study (*), or by differential scanning calorimetry in our previous report (#).^[21c]^

## Results

### Cremonese Spruces Remain Structurally Intact

**Figure 1:**
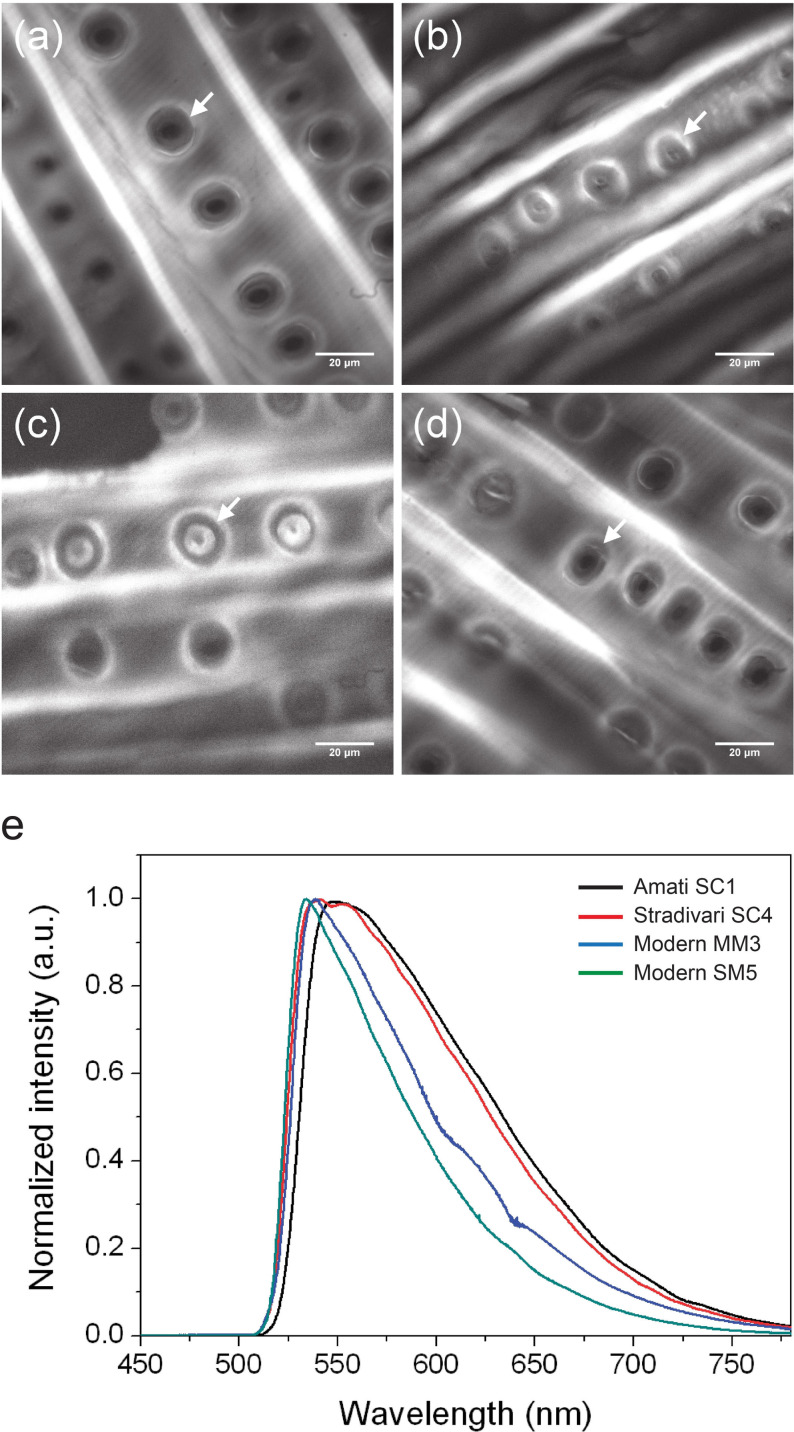
Optical‐sectioning hyperspectral images of spruces: a) Amati SC1, b) Stradivari SC4, c) modern SM3, and d) modern SM5, showing tracheid cells with bordered pits (arrows). e) The broader autofluorescence peaks of Amati and Stradivari suggest chemical heterogeneity due to lignin oxidation.

**Figure 2:**
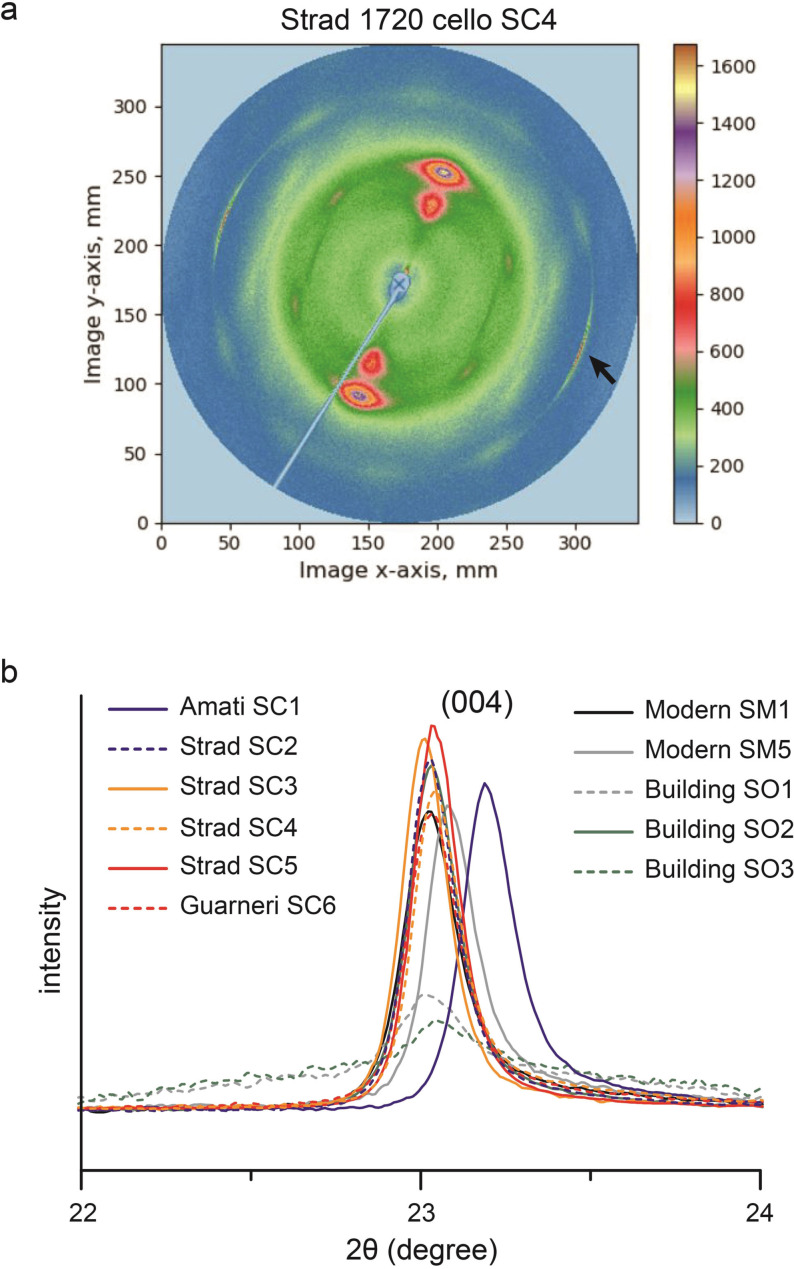
a) XRD patterns of Stradivari's spruce (SC4), with the arrow labeling the (004) peak. b) The (004) peaks of Cremonese, old, and modern spruces. Old building samples SO1 and SO3 exhibit weaker peaks, suggesting reduced crystallite length.

The visual appearances of Cremonese spruces are slightly more yellow than modern specimens (Supporting Information Figure S2), which is normal for aged wood due to the spontaneous oxidation of lignin.^[22]^ Cremonese wood cells show normal morphologies under optical‐sectioning hyperspectral imaging, and the broadening of lignin autofluorescence peaks likely reflects increased chemical heterogeneity caused by oxidation (Figure 1). Inspections using synchrotron X‐ray tomography (Supporting Information Figure S3–S5) and scanning electron microscopy (SEM) (Supporting Information Figure S6–S8) demonstrate that Cremonese spruces retained intact cellular and subcellular structures, including the fragile pit membranes. Some have proposed that fungal pre‐treatment of tonewood may bring acoustic benefits,^[23]^ but we did not observe fungal growth in Cremonese spruces. It has also been hypothesized that wood cellulose may slowly recrystallize over time,^[24]^ but we found no apparent differences in cellulose crystallinity between modern and Cremonese spruces, as their X‐ray diffraction peaks appear mostly identical (Figure 2 and Supporting Information Figure S9). Crystallite dimensions calculated using the Scherrer equation also remain unchanged (Supporting Information Table S4). By contrast, some spruce specimens from 18th‐century buildings show much weaker (004) diffraction peaks and shorter crystallite lengths (Figure 2), possibly damaged by weight loading and weathering.[^22^, ^25^] Fortunately, spruce woods from Stradivari and Guarneri instruments retained structural integrity even after three centuries of playing.

### Cremonese Spruces Exhibit Unusual Oxidation Patterns

**Figure 3:**
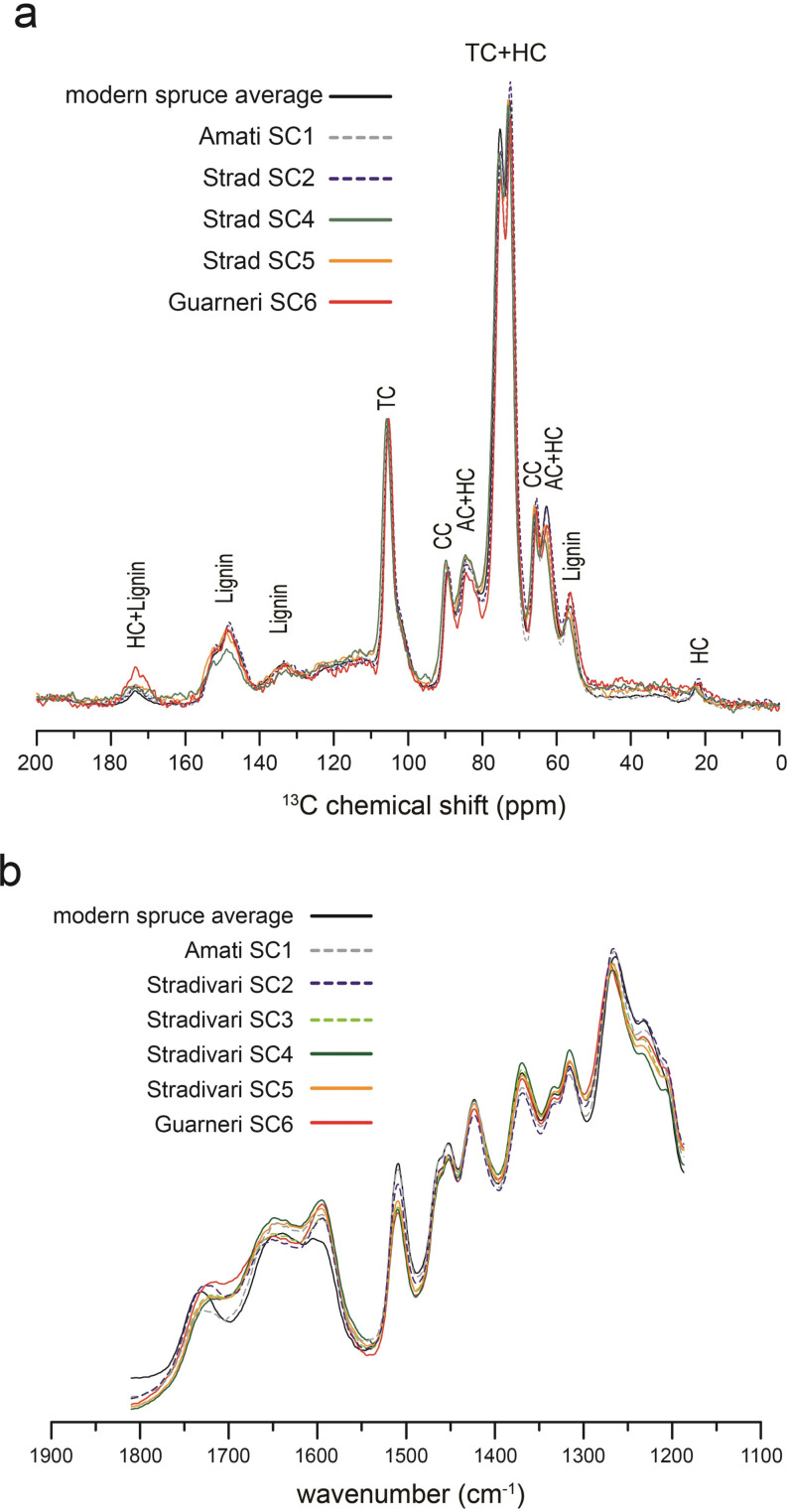
a) ^13^C{^1^H} multiCP ssNMR spectra of Cremonese spruces, compared to the average of modern spruce controls (SM1–SM5). HC: hemicellulose. TC/AC/CC: total/amorphous/crystalline cellulose. b) Infrared absorption spectra of Cremonese spruces.

Next, we examined the effects of aging on Cremonese spruces using ^13^C solid‐state nuclear magnetic resonance (ssNMR; Figure 3 a and Supporting Information Figure S10, S11), and they show little sign of degradation. By contrast, Cremonese maples show significant hemicellulose degradation and lignin demethoxylation (Supporting Information Figure S12), comparable to our previous observations.^[21c]^ Such distinctions may be caused by fundamental differences in wood chemical compositions. The basic building blocks of hemicellulose and lignin are very different between conifers/softwoods (e.g. spruce) and dicots/hardwoods (e.g. maple).^[26]^ Liquid chromatography measurements indicate that maple carries three times higher hemicellulose acetylation than spruce (Supporting Information Table S5), which may slowly transform into acetic acid during aging to catalyze hemicellulose hydrolysis.[^21c^, ^27^] The Guarneri spruce shows a stronger peak at 175 ppm (carbonyls) compared to other samples, which may reflect oxidative modification (Figure 3 a).

Spruces aged for 200–400 years exhibit decreased infrared absorption at 1735 cm^−1^ (Supporting Information Figure S13–S15), caused by hemicellulose deacetylation.[^21a^, ^28^] This becomes even more evident in aged softwood taken from millennium‐old Chinese zithers (Supporting Information Figure S16). Interestingly, Stradivari and Guarneri spruces uniquely exhibit increased absorption around 1710 cm^−1^ (Figure 3 b), implicating the generation of ketones or carboxylic acids via oxidation.^[29]^ We tested several common wood treatments—boiling, alkaline solutions, ultraviolet radiation, and baking—and found the latter two to enhance absorption at 1710 cm^−1^ (Supporting Information Figure S17). It is plausible that Stradivari and Guarneri had exposed their soundboards to strong sunlight, as described in Stradivari's personal letter,^[5]^ but we cannot rule out the possibility of baking or more complex chemical procedures. The absence of protein signature peaks in infrared spectra confirmed that Cremonese wood samples were not contaminated by collagen glues used for violin assembly and repairs.

### Cremonese Spruces Contain Mineral Additives

**Figure 4:**
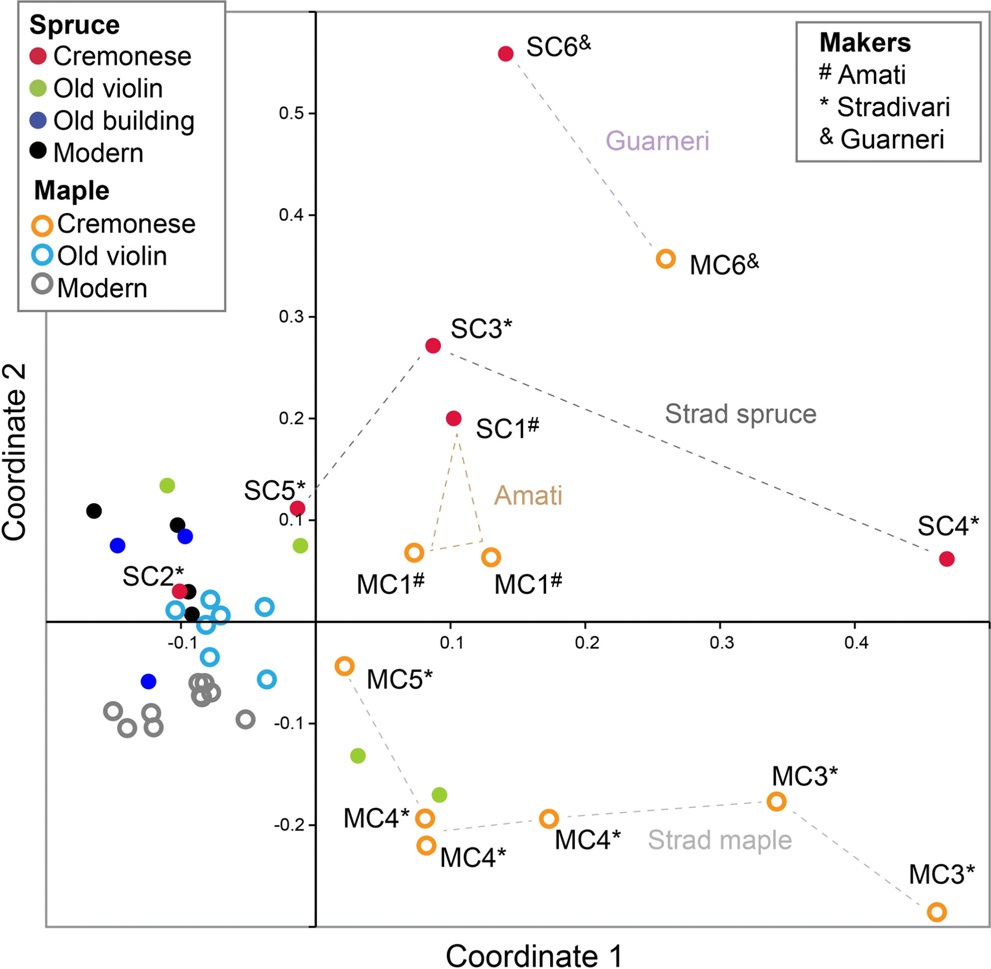
Elemental profiles of Cremonese spruces and maples measured by ICP‐MS, compared to modern and age‐matched controls, represented by multi‐dimensional scaling analysis.

To search for evidence of chemical manipulation, inductively coupled plasma mass spectrometry (ICP‐MS) was used to quantify over two dozen elements in Cremonese spruce and maple specimens by digesting wood chips in nitric acid. The controls included modern and age‐matched tonewoods, as well as chemically treated woods. The complete elemental measurements are listed in Supporting Information Table S6–S13 (also provided as spreadsheet file in Supporting Information). To understand possible contamination from human contact or unscrupulous repairs, we included four unexceptional old violins (18th–19th centuries) with extensive repairs and intentionally sampled their wood from the body‐contact areas. These areas showed elevated K, Na, and Cl levels, indicative of human sweat.^[30]^ Against these background references, we identified additional elemental changes that may be attributed to Cremonese manipulation, as summarized in Table 1. The results are graphically presented using multidimensional scaling (Figure 4) and principal component analysis biplots (Supporting Information Figure S18). The elemental profiles of Cremonese spruce and maple specimens are rather distinct from those of control groups, except for sample SC2 (Figure 4). Although the MC5 chip (1731 Stradivari cello) analyzed in this study appears to be lightly treated, two other chips from this specimen showed much more mineral additives in our previous study.^[21c]^ It implies that the original mineral treatment was not fully penetrating and that the ions cannot freely diffuse in dry wood even after centuries. Hence, we cannot distinguish whether sample SC2 originated from an untreated plate or the impenetrable region of a treated plate.

The first reported elemental analysis of Cremonese spruce found 700 ppm of Al for an Andrea Guarneri cello.^[31]^ In this study, we found little Al (<50 ppm) in two samples from his teacher Amati or eight samples from his neighbor Stradivari. But both samples from his grandson contained high Al levels (1200 and 2900 ppm). This is most likely attributed to experimentations involving family recipes instead of purchasing wood pre‐treated by lumber suppliers or having random contaminations during subsequent centuries. Thus, experimentation along different directions and location‐dependent heterogeneity may explain the observed variabilities among Stradivari samples.

From these elemental changes, we deduced the corresponding mineral candidates by referring to historically available materials.^[32]^ Nicolo Amati's early recipe likely contained low levels of borax (Na_2_B_4_O_7_) and metal sulfates (Cu/Fe/Zn), which may serve as fungicides or insecticides.^[33]^ These preservatives were similarly applied by Amati's pupils, the Guarneri and Stradivari families. High levels of Al and Ca in Guarneri samples probably represent alum (KAl(SO_4_)_2_, NH_3_Al(SO_4_)_2_, or Al_2_(SO_4_)_3_) and lime (Ca(OH)_2_), respectively. Some Stradivari samples also contain low levels of Al. Certain Stradivari samples (SC4, MC3, and MC4) contain exceedingly high levels of Na, Cl, and K, higher than other old instruments by thousands of ppm. This probably involved salt seasoning (NaCl), an ancient practice to prevent cracking by retaining moisture under dry weather.^[34]^ The source of K may have been plant‐derived potash (K_2_CO_3_ and KOH). Mild alkaline treatments using potash or lime may help remove organic extractives (resin acids, tannic acids, etc.) and nutrients (sugars, oils, etc.) to prevent discoloration and biological attacks.^[35]^ The samples analyzed for this study were removed from the unvarnished interior side of plates, but it remains possible that some elements may have diffused from the varnish into the wood at low concentrations.

### Altered Cellulose Nanostructure in Stradivari Spruce

**Figure 5:**
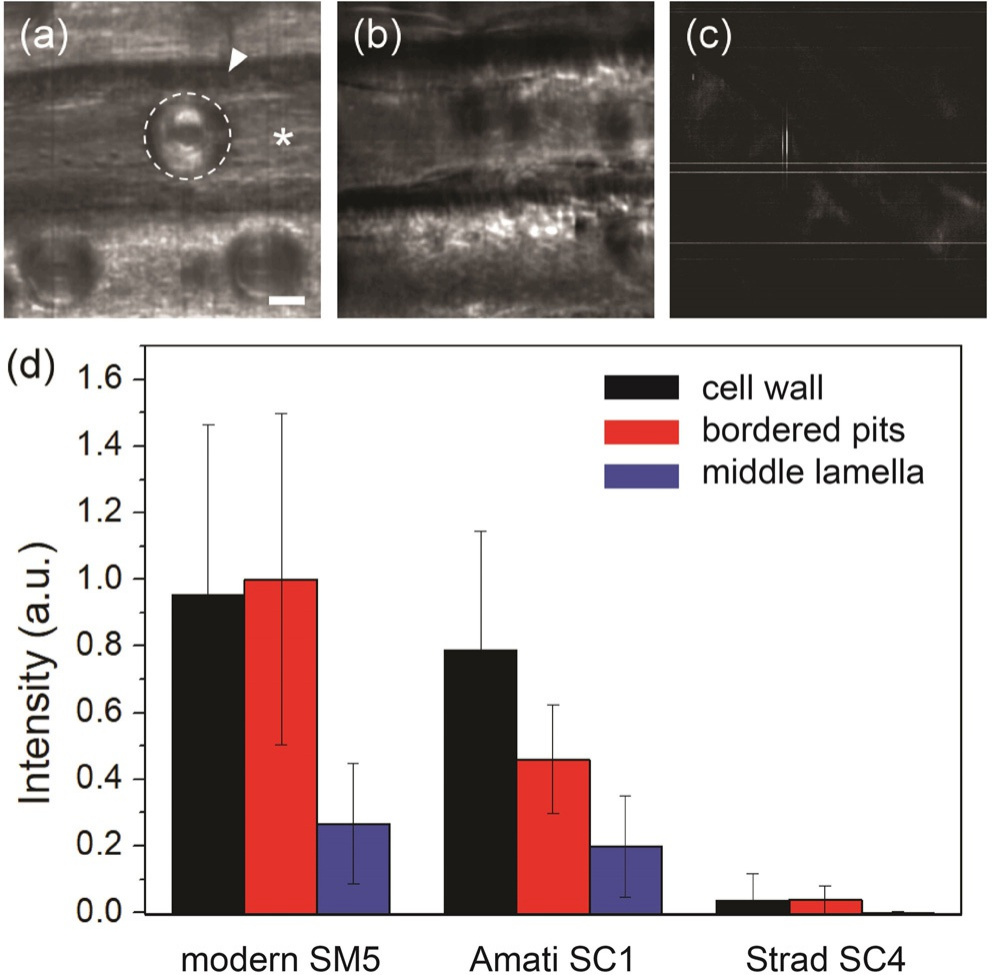
SHG images of a) modern, b) Amati, and c) Stradivari spruce sample. The visible structures include tracheid cell walls (star), bordered pits (dashed circle), and middle lamella (arrowhead). Scale bar=10 μm. Straight lines in (c) are camera detector artifacts that become visible after image enhancement. d) SHG intensities of different regions in different samples, with error bars representing the standard deviation.

As shown in Figure 4, Stradivari SC4 and Guarneri SC6 received the most extensive chemical treatments among Cremonese spruces. When two‐photon hyperspectral imaging^[36]^ was used to examine the cellular structure of SC4, we made the surprising observation that second‐harmonic generation (SHG) signals have largely diminished, and the results are quantified in Figure 5. In contrast, the Amati SC1 sample, despite being older and mildly treated with chemicals, still shows strong SHG signals. SHG is a non‐linear optical phenomenon associated with non‐centrosymmetry, arising from cellulose microfibrils with helical twists.^[37]^ Diminished SHG suggests conformational changes and rearrangements of cellulose chains. This may be caused by the aggregation of cellulose microfibrils, which can only occur after the partial removal of surrounding hemicellulose chains.[^37^, ^38^] In Figure 3 b, SC4 shows severely reduced infrared absorption at 1230 cm^−1^, consistent with alkaline extraction of hemicellulose^[39]^ (also see Supporting Information Figure S17). Similarly, cellulose rearrangement and hemicellulose decomposition have also been reported for Stradivari maples MC3 and MC4.^[21c]^ We noticed that SC4, MC3, and MC4 received similar and extensive chemical treatments (Table 1), with high K levels to suggest potash application for fragmenting hemicellulose.

### Aluminum Crosslinking in Guarneri Spruce

**Figure 6:**
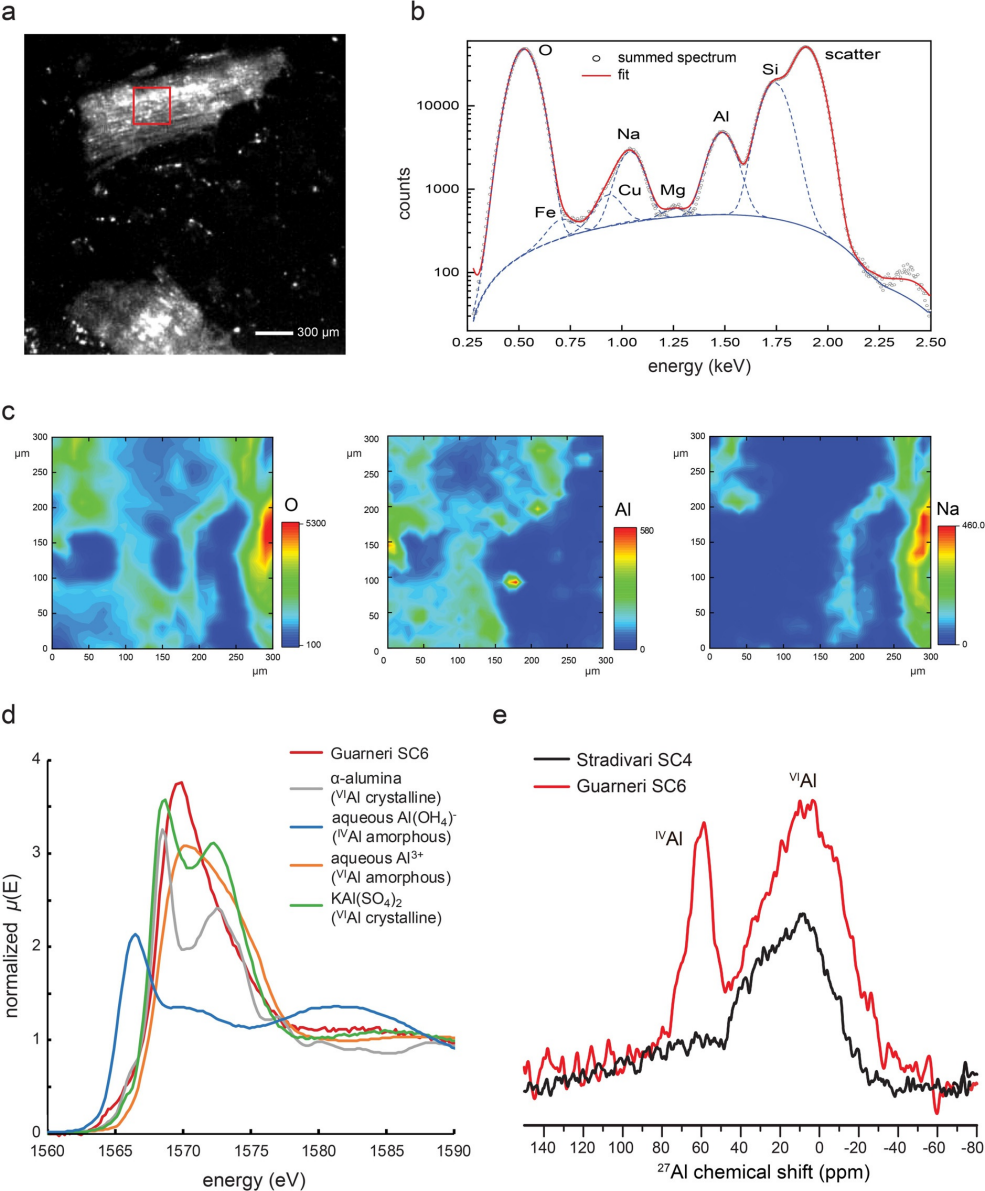
a) Brightfield microscope image of Guarneri spruce (SC6). The red box is analyzed for XRF. b) Cumulative XRF spectra with curve fitting for different elements. c) Spatial mapping of individual elements by XRF. d) XANES spectra of Guarneri spruce compared to standards. e) ^27^Al ssNMR spectra of Guarneri and Stradivari spruces.

High concentrations of Al ions have the potential to crosslink wood polymers via chelation. The chelating ligands may include hydroxy, carbonyl, phenolic, and carboxylate groups present on cellulose, hemicellulose, and lignin polymers.^[40]^ By synchrotron X‐ray fluorescence (XRF) imaging, we found Al to be diffusely distributed throughout the wood matrix of Cremonese spruces SC4 and SC6 (Figure 6 a–c and Supporting Information Figure S19). The X‐ray absorption near edge structure (XANES) spectrum of Al is consistent with chelated (amorphous) states instead of crystalline states (Figure 6 d). The coordination state is further investigated using ^27^Al ssNMR (Figure 6 e). We observed mostly six‐coordinate Al species (^VI^Al, approx. 0 ppm) in Stradivari and Guarneri specimens, but the latter also contained some ^IV^Al species (approx. 60 ppm). In comparison, alum treatment of modern spruce only resulted in ^VI^Al species, but very minor conversion to ^IV^Al may occur after immersion into alkaline buffers (Supporting Information Figure S20, S21). The unusual presence of ^IV^Al in Guarneri spruce may reflect elevated pH inside the wood due to alkaline treatments. Al^3+^ tends to transition from 6‐ to 5‐ and then to 4‐coordinate state as the pH goes from acidic to alkaline.^[41]^

Given the diminutive size of Cremonese wood shavings available for study, it is not feasible to determine their mechanical or acoustic properties. To understand the potential effects of metal crosslinking, we introduced into cellulosic headphone diaphragms 1000 and 3000 ppm of Al ions (to simulate the levels found in Guarneri's spruce and maple, respectively) and found augmented low‐frequency response below 500 Hz (Supporting Information Figure S22). Moreover, Young's modulus also increases 14 % with 1000 ppm Al (Supporting Information Figure S23). The application of 3 % alum solution through f‐holes had also been reported to alter the plate resonance of violins.^[18]^ It is plausible that Al^3+^ crosslinking may exert acoustic effects by increasing the stiffness‐to‐weight ratio of violin plates. Moreover, it has been reported that salt seasoning of fir (30 000 ppm Na) raised Young's modulus by 19 % and crushing strength by 33 %.^[42]^ This may be due to the lowering of water activity by salt, altering non‐covalent bonding between wood fibers.^[43]^ Thus, we cannot rule out the possibility of salt‐induced stiffening in Stradivari specimens SC4 (13 000 ppm Na) and MC3 (11 000 ppm Na). Modern violin makers may consider testing additional trivalent and tetravalent metal ions such as La^3+^ or Zr^4+^. Surprisingly, Guarneri's maple already contained 40 ppm of Zr^4+^ (Supporting Information Table S8), half a century before the element was discovered.^[44]^

## Discussion

Natural aging is known to alter the mechanical properties of wood and likely to affect the acoustic output of violins.[^24^, ^45^] However, aging alone cannot explain the unique qualities of Stradivari and Guarneri violins, because thousands of other master violins from the same period never gained similar appreciation from soloists. Aging also increases the risk of wood cracking due to repeated dimensional changes and creeping, caused by temperature and humidity fluctuations.^[46]^ The fact that Stradivari and Guarneri spruces can be carved extra thin and last for centuries implies that chemical modifications have enhanced their durability. Our data suggest several plausible protective mechanisms: (a) Prevention of fungal and worm damage; (b) Adding alum to crosslink and stabilize wood polymers; (c) Adding NaCl to prevent excessive shrinkage in dry weather;^[34]^ (d) Wood stiffening by alum or NaCl;^[42]^ (e) Applying mild alkaline treatment for partial fragmentation of hemicellulose,^[47]^ the most hygroscopic component of the cell wall,^[48]^ to reduce moisture absorption and improve dimensional stability. Ancient Chinese zither makers also advocated alkaline lime treatment for the artificial aging of wood.^[49]^

The cellular and ultrastructural features of Cremonese spruces closely resemble those of modern spruce used by violin makers, suggesting similar botanical origins. We did not find any apparent anomalies or damages in Cremonese samples, as examined by SEM, XRD, X‐ray tomography, and hyperspectral imaging. There have been puzzling CT reports showing that density differentials between earlywood and latewood are unusually small in Cremonese spruces but not maples.^[20b]^ Judging from our data, it is probably an artifact caused by the preferential absorption of metal salts into earlywood, causing greater X‐ray absorption and an overestimation of wood density. The tree rings of spruce are clearly visible and the earlywood is more porous and less dense compared to latewood. In contrast, the tree rings of maple are not clearly visible and no anomaly is reported.

We do not yet understand the details of wood treatment processes employed by Cremonese makers. Based on discussions with experienced violin makers, we have proposed a plausible scheme. Cremonese masters probably purchased untreated tonewood from local suppliers,^[3b]^ either air‐dried or semi‐wet. In individual workshops, the tonewood may have been immersed into various solutions containing table salt, lime, potash, alum, etc. Our analysis cannot determine if the wood was also boiled or steamed at some stage to kill fungi or worms. In the ensuing drying phase (air‐dried or heated), fungicidal preservatives like borax and sulfates of Cu/Fe/Zn were likely applied to the surface. Solutions applied to the surface of dried spruce hardly penetrate beyond 0.2 mm, but our samples originated from 0.5 mm or deeper. After assembly and varnishing, the instrument was exposed to sunlight to polymerize the varnish, as described by Stradivari.^[5]^ The combination of redox‐active metals (Cu and Fe),^[50]^ heat,^[51]^ or ultraviolet radiation^[52]^ could possibly lead to the unusual oxidation patterns observed by infrared and NMR spectroscopy. Moreover, the wood aging process could be affected by these initial treatments. The chemical and physical interactions involved may be very complex and need to be further investigated.

The thickness of violin soundboards is an important parameter for violin acoustics.[^11b^, ^53^] The thinner soundboards in Stradivari and Guarneri violins are thought to enhance sound intensities in the 2–4 kHz range relative to the 4–6 kHz range—adding brilliance and reducing harshness.^[54]^ However, this effect may be associated with the special properties of Stradivari's and Guarneri's woods. Artificial modification of tonewood may exert complex effects on mechanical properties. For instance, vibrational damping (loss tangent) can be affected by changes in moisture absorption and displacement, which are in turn affected by hemicellulose breakdown or mineral additives.[^24^, ^34^, ^55^] Modulus of elasticity may also be affected by hemicellulose breakdown, metal crosslinking, or salt seasoning.[^25^, ^34^, ^42^] As shown in Table 1, Stradivari samples that received extensive chemical treatments exhibit signs of cellulose rearrangement. How this affects mechanical properties needs to be further investigated. Changes in the mechanical properties of wood could exert significant effects on violin acoustics, comparable to changing basic physical constraints such as geometry,^[15c]^ but there is a lack of computational models that can accurately predict such effects.[^1^, ^16a^, ^56^]

Stradivari instruments have served musicians for over three centuries, and many wonder how much longer they may last. Recent studies have shown that an Amati violin made 450 years ago still produces tones similar to those of new violins.^[9]^ However, the half‐life of maple hemicellulose has been estimated to be approximately 400 years,^[21c]^ and its spontaneous breakdown will eventually cause structural failures and tonal degradation. We cannot estimate the half‐life of spruce hemicellulose in this study because of its very slow decomposition rate. It appears to be even longer than the estimate of approximately 800 years given by Pishik et al.,^[57]^ possibly because our samples are better preserved. We predict that aging will cause cell wall breakdown in the maple before the spruce and that the service life may be extended by replacing maple backs and ribs. The ideal replacement material may be aged maple that has been chemically treated in similar ways as the original.

## Conclusion

For the first time, solid evidence has been gathered to validate the hypothesis that soundboard materials used by Stradivari and Guarneri differ significantly from the unprocessed spruce preferred by modern makers. This study tracked the evolution of wood engineering in Cremona for over 120 years (1619–1741). Chemical additives were applied to Cremonese soundboards as well as back plates—likely intended for wood preservation and acoustic tuning. The Amati recipe was relatively simple but his pupils, the Stradivari and Guarneri families, developed much more complex formulations. The consequences of chemical treatments may include hemicellulose fragmentation, altered cellulose nanostructures, and metal crosslinking. Further experimentation with various mineral recipes on assembled violins will be required to understand the acoustic effects of wood modification.

It is unfortunate that we have been searching for the secrets of Stradivari for over two centuries without realizing that the crucial soundboard is made of specially engineered wood, due to the lack of any historical clues. The materials engineering paradigm rediscovered in this study may explain why it has been so challenging to reproduce these acclaimed instruments. Our findings provide new experimental directions for violin making in the 21st century, which may also extend to other instruments incorporating spruce soundboards—pianos, harps, guitars, lutes, harpsichords, etc. There is little reason to believe that 18th‐century Italian craftsmanship cannot be reproduced or even surpassed with the aid of modern science.

## Materials and Methods

### Wood Samples

Wood samples listed in Supporting Information Table S1 and S2 were generously provided by Chimei Museum, Rene Morel, Guy Rabut, David Hume, John Harte, Melvin Goldsmith, Sandro Chiao, and Boa‐Tsang Lee. Unexceptional old European violins were purchased from Mathias Renner. Wood from a fine‐sounding antique Chinese guqin (7‐string zither) was kindly provided by Kin Woon Tong. Modern Chinese fir was provided by Dan Lu.

Cremonese wood specimens were originally removed during professional restorations without causing unnecessary damages, but often limited in quantity (30–300 mg). Samples from Cremonese violins and antique Chinese guqins were taken from the interior of top and back plates (unvarnished sides) by professional restorers, except for MC4 (stand‐alone Stradivari neck) that was carved out in the Tai laboratory. Cremonese spruce and maple samples were generally taken from a depth of 0.5 mm or greater. Surface layer materials (<0.3 mm) were not collected because they developed darker colors and may carry surface contaminations. For unexceptional old violins, we intentionally sampled wood from body contact areas to understand contact contamination. These included spruce from the chinrest area (SA1–SA4) and maple from the shoulder rest area (MA1–MA3, MA5), sampling the wood right beneath varnish by scraping away the varnish first. These were inexpensive violins from 18th–19th centuries with little practical value because they exhibited multiple repairs and serious structural damages. See Supporting Information Tables S1–S3 for further details.

### Synchrotron X‐ray Diffraction

The experiments were performed at the BL01C2 beamline of the National Synchrotron Radiation Research Center (Hsinchu, Taiwan), in which the ring was operated at 1.5 GeV energy with a typical current around 360 mA. A thin slice of the wood sample was fixed with 3 M Magic Tape and placed on the sample stage. Two pairs of slits and one collimator were set up to provide a collimated beam with dimensions of 0.1×0.1 mm (H×V) at the sample. The wavelength of the incident X‐rays was 1.033210 Å (12 keV), delivered from the 5‐T Superconducting Wavelength Shifter and a Si(111) triangular crystal monochromator. The scattering signal was collected with a Mar345 imaging plate area detector, setting the exposure duration at 30 s. The diffraction angles were calibrated according to Bragg positions of CeO_2_ (NIST SRM 674b) standards in desired geometry, and then GSAS II software (Argonne National Laboratory) was used to obtain a corresponding one‐dimensional powder diffraction profile with cake‐type integration. The profile data were exported to Origin2015 software for baseline correction and smoothing. The size of the crystalline domain (*L*) is estimated using the Scherrer equation [Eq. 1], where *K* is the shape factor equal to 0.94 for wood cellulose,^[58]^ *λ* is the X‐ray wavelength, *B* is the full width at half maximum in 2*θ* units, and *θ* is the Bragg angle.

(1) L=Kλ=Bcosθ

### ICP‐MS

Around 10 mg to 20 mg of finely cut wood sample were weighed and digested with 3 mL of 70 % (wt/wt) HNO_3_ (ultrapure reagent grade) in a 10 mL perfluoroalkoxy alkane vial precleaned with 30 % (wt/wt) HNO_3_. The digestion mixture was appropriately diluted with 0.5 % HNO_3_ and analyzed by Agilent 7700x ICP‐MS (Santa Clara, CA). Standard solutions for calibration curves were prepared from dilution of high‐purity standards of individual elements (1000 mg L^−1^) in 0.5 % HNO_3_. ICP‐MS was operated at 1.5 kW radio frequency (RF) power and reaction mode (He, 4.0 mL min^−1^), with plasma/auxiliary/carrier/makeup flow rates at 15/0.9/1.0/0.1 L min^−1^. Quantification was based on the signal intensities of *m*/*z* 11 (B), 23 (Na), 24 (Mg), 27 (Al), 28 (Si), 31 (P), 34 (S), 35 (Cl), K (39), 42 (Ca), 47 (Ti), 53 (Cr), 55 (Mn), 57 (Fe), 60 (Ni), Cu (65), Zn (66), 75 (As), 82 (Se), 88 (Sr), 90 (Zr), 111 (Cd), 118 (Sn), 121(Sb), Ba (137), Hg (202), and Pb (208) with 0.3 s integration time and in three replicates. The accuracy was validated by taking measurements of Standard Reference Material 1575A (trace elements in pine needles; National Institute of Standards and Technology) with each batch of samples, and by comparing to the published reference values (Supporting Information Table S13).^[59]^

## Conflict of interest

The authors declare no conflict of interest.

## Supporting information

As a service to our authors and readers, this journal provides supporting information supplied by the authors. Such materials are peer reviewed and may be re‐organized for online delivery, but are not copy‐edited or typeset. Technical support issues arising from supporting information (other than missing files) should be addressed to the authors.

Supporting Information

Supporting Information
