# Supplementary material for: Materials Engineering of Violin Soundboards by Stradivari and Guarneri
Source: Angew Chem Int Ed Engl. 2021 Jun 27;60(35):19144–54. doi: 10.1002/anie.202105252 (PMC8457145; doi:10.1002/anie.202105252)
Supplement: Supplementary file 2 — Supporting Information [file ANIE-60-19144-s001.pdf]

Angewandte Chemie
Eine Zeitschrift der Gesellschaft Deutscher Chemiker

Supporting Information

Materials Engineering of Violin Soundboards by Stradivari and Guarneri

*Cheng-Kuan Su+, Szu-Yu Chen+, Jen-Hsuan Chung, Guo-Chian Li, Brigitte Brandmair, Thomas Huthwelker, John L. Fulton, Camelia N. Borca, Shing-Jong Huang, Joseph Nagyvary, Hsiao-Han Tseng, Chih-Hui Chang, Dai-Ting Chung, Rafael Vescovi, Yi-Shiuan Tsai, Wenjie Cai, Bing-Jyun Lu, Jia-Wei Xu, Chia-Shuo Hsu, Jun-Jie Wu, Hao-Zhi Li, Yu-Kai Jheng, Sheng-Fong Lo, Hao Ming Chen, Yi-Ting Hsieh, Po-Wen Chung, Chien-Sheng Chen, Yuh-Chang Sun, Jerry Chun Chung Chan, and Hwan-Ching Tai**

anie_202105252_sm_miscellaneous_information.pdf
anie_202105252_sm_ICPMS.xlsx

Author Contributions

CKS, SYC, JHC, GCL, TH, JLF, CNB, SJH, HHT, HCH, RV, YST, BJL, JWX, CSH, JJW, HZL, and YKJ performed laboratory measurements and compiled the data. SYC, SFL, HMC, YTH, PWC, CSC, YCS, JCCC, and HCT conceived the experiments and analyzed the data. BB, JN, DTC, and WC provided historical samples and historical information. HCT wrote the manuscript with input from all authors.

Supporting Information

Table of Contents

I. Materials and Methods 4

II. Supplementary Tables 10

Table S1. Wood samples from antique instruments 10

Table S2. Wood samples from old and new tonewood planks 11

Table S3. Characterization of wood from an antique Chinese guqin 12

Table S4. X-ray diffraction data of modern and historical maples 13

Table S5. Microchemical analysis of modern spruce and maple tonewood 14

Table S6. ICP-MS elemental analysis of antique violin and Cremonese spruces 15

Table S7. ICP-MS elemental analysis of modern and old building spruces 16

Table S8. ICP-MS elemental analysis of Cremonese maples 17

Table S9. ICP-MS elemental analysis of modern maples 18

Table S10. ICP-MS elemental analysis of antique violin maples 19

Table S11. ICP-MS elemental analysis of chemically treated spruces 20

Table S12. ICP-MS elemental analysis of chemically treated maples 21

Table S13. ICP-MS analysis of NIST Standard Reference Material 1575a 22

IV. Supplementary Figures 23

Figure S1. Three major violin making families during the Golden Age of Cremona 23

Figure S2. Photographs of Cremonese and control spruce samples 24

Figure S3. Volume rendering of X-ray tomography data for modern spruce 25

Figure S4. Volume rendering of X-ray tomography data for Amati spruce 26

Figure S5. Volume rendering of X-ray tomography data for Stradivari spruce 27

Figure S6. SEM image of modern spruce 28

Figure S7. SEM images of Amati spruce 29

Figure S8. SEM images of Stradivari spruce 30

Figure S9. X-ray diffraction peaks of (200) for modern and historical spruce samples 31

Figure S10. $^{13}C\{^{1}H\}$ multiCP NMR spectra of modern spruces 32

Figure S11. $^{13}C\{^{1}H\}$ multiCP NMR spectra of spruce specimens from old buildings 33

Figure S12. $^{13}C\{^{1}H\}$ multiCP NMR spectra of Cremonese maple specimens 34
Figure S13. Infrared absorption spectra of modern spruce specimens 35
Figure S14. Infrared absorption spectra of spruce specimens taken from old buildings 36
Figure S15. Infrared absorption spectra of spruces from unexceptional old European violins 37
Figure S16. Infrared absorption spectra of firs from antique Chinese zithers...38
Figure S17. Infrared absorption spectra of modern spruce after artificial treatments 39
Figure S18. Principal component analysis of elemental concentrations of spruce and maple 40
Figure S19. 2D mapping of elemental distributions in Stradivari spruce..41
Figure S20. ^{27}Al solid-state NMR spectra of aluminum model compounds 42
Figure S21. ^{27}Al solid-state NMR spectra of alum/alkaline-treated spruce 43
Figure S22. The effects of aluminum impregnation on acoustic responses 44
Figure S23. The effects of aluminum impregnation on Young's moduli..45

V. References...46

I. Materials and Methods

Reagents and Chemicals

$KAl(SO_4)_2$ (alum), K_2CO_3, 4-(2-hydroxyethyl)-1-piperazineethanesulfonic acid (HEPES), and N-cyclohexyl-3-aminopropanesulfonic acid (CAPS), 1-butyl-1-methylpyrrolidium dicyanamide (BMP-DCA), and methyl propylene glycol ether were purchased from Sigma (St. Louis, MO). Oak ash was purchased from Chirori (Osaka, Japan). Ultrapure reagent grade HNO_3 was purchased from Baker (Waltham, MA).

Artificial wood treatments

Wood samples were cut into thin flakes (30-50 mg each) and immersed into treatment solutions for 72 h at r.t., followed by three rinses with deionized water using spin cups with cellulose acetate filter (Thermo, Waltham, MA), and stored in a desiccator cabinet (~40% relative humidity). The treatment solutions included KOH (pH 11.5), $Ca(OH)_2$ (pH 11), 5% NaCl (50 g/L), 1% $KAl(SO_4)_2$ (10 g/L, pH 3.4), and 2% K_2CO_3 (20 g/ L, pH 11.6). Lye treatment solution (pH ~10.4) was prepared by dissolving 0.1% oak ash in water and filtered through filter paper and 0.22 μM Steriflip-GP filter (Millipore, Burlington, MA).

To simulate a combination of alum and alkaline treatment, wood flakes treated with 1% $KAl(SO_4)_2$ were further immersed for 6 h in alkaline buffers of different pH values: pH 8 (50 mM HEPES), pH 9 (50 mM HEPES), pH 10 (50 mM CAPS), and pH 11 (50 mM CAPS). After three rinses with deionized water using spin cups with cellulose acetate filter (Thermo, Waltham, MA), the samples were stored in a desiccator cabinet (~40% relative humidity).

Baking of wood was conducted by placing thin flakes in an oven for 3 h under normal atmosphere at 200 ^{0}C. Boiling of wood was conducted by placing thin flakes in a deionized water for 72 h at 95 ^{0}C. Ultraviolet (UV) radiation treatment was conducted by illuminating thin wood flakes using a xenon lamp solar simulator for 24 h at r.t. (AG 1.5, 100 mW/cm^2).

Radiocarbon dating

Wood samples (10-20 mg) were pretreated by acid-base-acid method [1]. The sample was subjected to boiling in 1 M HCl for 1 h, rinsing several times with deionized water, washing with 0.5 M NaOH (70 °C), rinsing several times with deionized water, boiling in 1 M HCl for 1 h, rinsing several times with deionized water, and oven-drying. The samples were analyzed by National Taiwan University Accelerator Mass Spectrometer (NTU AMS) ^{14}C Dating Laboratory, using an HVE 1.0 MV Tandetron 4110BO instrument (Amersfoort, Netherlands). Calendar age was calculated from the ^{14}C age using the CALIB 7.10 program [2], based on IntCal 13 calibration curve [3].

Wood composition analysis

The wood sample was granulated and sieved below 120 μm. The glass microfiber filter paper (Whatman, Maidstone, UK) was thermally pretreated in a muffle furnace at 575 ˚C for 3 h. Dried wood sample (50 mg) was incubated with 72% (w/w) sulfuric acid (0.5 mL) in a pressure tube (Hungate tube)

at r.t. and vortexed every 15 min. After 1 h, deionized water (14 mL) was added and autoclaved at 121 ˚C for 1 h. Afterwards, the mixture was stored in at 4 ˚C overnight. The supernatant was filtered with syringe filter (polytetrafluoroethylene membrane, 0.22 µm), and analyzed via high-performance liquid chromatography (HPLC) to determine the glucose, xylose, mannose, arabinose, and acetyl content. The acid-insoluble solid was filtered through the pretreated glass microfiber filter paper, rinsed with deionized water until reaching neutral pH value, and dried at 105 ˚C overnight. The dried sample was weighed (m_1) and calcined at 575 ˚C for 3 h. The weight of the ash was also determined (m_2). The Klason lignin content was calculated by the mass difference between the dried solid (m_1) and ash (m_2).

For HPLC, the samples were eluted at 50 ºC through an Aminex HPX-87H column (300 x 7.8 mm, Bio-Rad, Hercules, CA) on a Shimadzu Prominence LC-20AD LC system (Kyoto, Japan) equipped with a RID-20A refractive index detector. The elution was performed with 0.01 N H_2SO_4 at a flow rate of 0.6 mL min^{-1}. The determined monosaccharide content was converted to polysaccharide content using the anhydro correction factor of 0.9 (glucan and mannan) or 0.88 (xylan and arabian).

Scanning electron microscopy

The examination of archaeological wood using ionic-liquid treatment and scanning electron microscope (SEM) follows recently reported procedures [4]. The wood sample was immersed into the 7.5 vol% BMP-DCA ionic liquid in ethanol solution for 30 s, and the excess ionic liquid was removed by tissue paper before being introduced into a JEOL JSM-IT100 instrument (Tokyo, Japan), operating at working distances of 10 mm with an accelerating voltage of 5 kV.

Synchrotron X-ray tomography

The tomography data shown was acquired at the 32-ID beamline [5] at the Advanced Photon Source (Lemont, IL). The setup consists of a λ_u = 1.8 cm period undulator operated at a low deflection parameter value of K = 0.26, so that a single quasi-monochomatic peak at E = 25 keV could be generated without loss due to optical elements. The sample was mounted on piezo stages on top of an air bearing rotary stage PI-Micos UPR-160 AIR (Auburn, MA). Tomographic projections were recorded by using a 10 µm thick LuAG:Ce scintillator to convert the propagation-enhanced X-ray intensity pattern into a visible light image which was then magnified using a microscope objective onto a visible light-scientific CMOS camera (Flir Grasshopper3, 1920x1200 pixels, Wilsonville, OR). Exposure times for a single projection image was set to 10 ms and each tomogram had 1800 rotation angles over 180 degrees of rotation. Data were stored on the experimental control computers at the beamline and processed using the cluster Cooley at the Argonne Leadership Computing Facility. Each acquisition was automatically processed using Automo [6] and Ripple [7]. The main reconstruction package used was TomoPy [8] and the reconstruction kernel used was the Gridrec implementation [9] of filtered backprojection, which offers rapid, non-iterative reconstructions. Larger areas were acquired at high resolution using the Tomosaic package [10]. The final reconstructions were rotated and cropped using python scripts and final rendering was done using the software Vaa3d [11].

Synchrotron XRF and XANES

The synchrotron-based micro-X-ray fluorescence (XRF) microscopy elemental mapping and subsequent micro-X-ray absorption spectroscopy (XAS) investigations have been performed at the

PHOENIX beamline of the Swiss Light Source (Villigen, Switzerland). The beamline provides monochromatic radiation in the soft and tender energy range (from 350 eV to 8000 eV). Source of the beamline is APPLE-II undulator (U54), which allows for both linear an elliptical polarization. For the measurements presented here linearly polarized light was used. The experiments were performed at the low energy branchline PHOENIX II, which is located at the exit of the X-Treme beamline [12]. The energy of the incident beam was selected using a planar grating monochromator. The monochromatic beam was focused down to a size of 10 μm using a Kirkpatrick–Baez (KB) mirror system.

The samples were attached by a conductive carbon tape to a copper plate. The plate was placed on the sample holder in the end station, which was kept under vacuum (10^{-6} mbar) and at room temperature. The sample was mounted at 45 degrees relative to both incident beam and fluorescence detector, which were mounted at an angle of 90 degrees relative to each other. This geometry minimizes elastic scattering contribution (Compton and Rayleigh). The intensity of the incoming beam, I_0, was measured as a total electron yield signal taken from a nickel coated polyester foil (0.5 μm thickness), located upstream of the KB system at a vacuum of about 10^{-7} bar. The X-ray fluorescence spectrum was recorded using an energy dispersive single-element silicon drift detector (KETEK GmbH, Munich, Germany) which provides an energy resolution of 150 eV. For the Al Ka fluorescence emission line (at 1570 eV), the attenuation length in cellulose is around 5 μm. Note that this probing depth is significantly higher than normally possible with the SEM at 5 kV accelerating voltages (around 100 nm for cellulose). Therefore, the elemental distribution and oxidation state of Al shown here are representative of the top layer of wood cells, with negligible influence of the surface selvedge region.

For the elemental maps, the incoming energy was fixed at 1900 eV, just above the Si K-edge such that several fluorescence lines could be recorded simultaneously for each pixel, including O, Na, Mg, Al, and Si. The maps were 300x300 μm in size and the pixel size was 10x10 μm. The dwell time of the detector was 2.4 s in each pixel. The elemental maps were generated from deadtime-corrected data and analyzed using Pymca software [13] using batch fitting of all spectra recorded for each map. The resulting fitted spectra were plotted separately for each element after background subtraction.

Several micro-XAS spectra at the Al K-edge were acquired for each sample based on the Al spatial distributions. The spectra were processed using the Athena software [14] by first normalizing against the incident flux (I_0), then to a post-edge value of 1. Each scan took around 12 minutes and the lowest energy step size used was 0.2 eV. The XAS spectra recorded with the micro-focused beam at different locations of the samples could reveal the Al oxidation state and its electronic structure. The shape of the Al K-edge spectrum changes significantly for four-fold compared to six-fold configurations of the surrounding oxygen atoms [15].

Infrared spectroscopy

Attenuated total reflection-Fourier transform infrared spectroscopy (ATR-FT-IR) was measured on Perkin Elmer Spectrum Two FT-IR spectrometer (Waltham, MA) and PIKE Technologies ATR accessory with ZnSe crystal (Fitchburg, WI). Wood samples were cut into small pieces and analyzed in absorbance mode with the following settings: wavenumber range of 650-4000 cm^{-1}, resolution of 4 cm^{-1}, and 32 scans for each spectrum. For each sample, six spectra were processed in the wavenumber range of 1187 to 1810 cm^{-1} by vector normalization and averaged by OPUS 7.5 software (Bruker, Billerica, MA). Vector normalization computes the average intensity of the selected wavenumber

range, and the value is subtracted from the spectrum to center the mean. Subsequently, the spectra are scaled to make the sum squared deviation over the indicated wavelengths equal to one.

Nuclear magnetic resonance spectroscopy

All solid-state nuclear magnetic resonance (NMR) experiments were carried out on a wide-bore 14.1-T Bruker Avance III spectrometer equipped with a 4-mm double-resonance magic-angle spinning (MAS) probe head. The Larmor frequencies for ^{1}H, ^{13}C, and ^{27}Al were 600.21 MHz, 150.94 MHz and 156.40 MHz, respectively. The spinning frequencies were controlled at 12 kHz. For multiple cross-polarization (MultiCP) MAS measurements, typical $\pi/2$ pulse lengths of 4 and 3.57 μs were applied for ^{13}C and ^{1}H, respectively. Proton decoupling field strength of $\gamma B1/2\pi = 75$ kHz was used. ^{13}C quantitative spectra were measured using the multiple-cross polarization (MultiCP) excitation method described by Johnson and Schmidt-Rohr [16], which offers better quantification than conventional CP experiments with fixed contact time. A total of nine CP blocks were implemented with 1 ms and RF amplitude increment (90–100%), while the last CP before the acquisition was executed with 0.8 ms and the same amplitude increment. The recycle delay was 2 s and the duration of the repolarization period t_z was 0.9 s. After the MultiCP excitation, a Hahn echo of two rotor periods was applied to achieve dead-time-free signal detection. ^{27}Al MAS NMR spectra were acquired with a single-pulse sequence with a central transition selective $\pi/4$ pulse of 0.8 μs and a recycle delay of 0.5 s.

Optical sectioning microscopy

The optical sectioning hyperspectral microscopy [17], based on a traditional confocal line-scanning hyperspectral system combined with structured illumination, was used to obtain both the 2D *x-y* optical sectioning images and 3D *x-y-λ* hypercubes of the specimens. In 2D mode, the 2D illumination, patterned with sinusoidal intensity distribution oriented at 45°, was generated by a DLP projector (LightCrafter, Texas Instruments, Dallas TX; 684*608 pixels). The light engine of this projector was an embedded blue LED with its spectral peak at 460 nm, which matched the excitation spectrum of the wood specimens. To achieve optical sectioning, a sequence of illumination patterns with different phases of 0, $2\pi/3$ and $4\pi/3$ was applied. A high numerical aperture objective (UPLSAPO 60XW, NA 1.2, Olympus Tokyo, Japan) projected the illumination pattern onto the wood specimen and collected the backward fluorescence signals from the specimen. The signals were separated from the excitation light by a dichroic beam splitter (FF509-FDi01, Semrock, Lake Forest, IL) and imaged onto an sCMOS camera (ORCA-Flash4.0 V2, Hamamatsu, Hamamatsu, Japan). In front of the sCMOS, a long-pass filter (BLP01-514R-25, Semrock) was inserted to block the excitation light and the background noises. For each optical sectioning image, three *x-y* patterned images had to be acquired for image reconstruction [18]. With 100X magnification, the pixel size of the image was 65 nm × 65 nm and the image size was 133 mm × 133 mm.

In 3D mode, the hyperspectral system was based on a line-scanning imaging system [17]. The excitation line was along the *y*-direction and the line was scanned along the *x*-direction. To achieve optical sectioning, each excitation line was patterned with sinusoidal intensity distribution and three excitation patterns with phases of 0, $2\pi/3$ and $4\pi/3$, as required for image reconstruction. The DLP projector was used to generate the sequence of the patterned excitation line. The objective was used to project the excitation light onto the wood specimen and collect the backward fluorescence signals from the specimen. The signals were separated from the excitation light by the dichroic beam splitter and

then guided into a home-built spectrometer. The line-shaped signals were first filtered by a slit and then collimated along the direction perpendicular to the slit (*l*-direction). The collimated signals hit the grating so that the spectra of the signals were dispersed spatially along the *l*-direction. The dispersed signals were imaged onto the sCMOS camera to get a y-λ spatio-spectral image. In front of the sCMOS, the long-pass filter blocked the excitation light and the background noises. To obtain a complete x-y-λ hypercube, a series of y-λ images were acquired by scanning the specimen along the *x*-direction with a translation stage. By setting 65-nm step distance for the *x*-direction scanning and a magnification of 100X for the *y*-direction imaging, the corresponding size at the sample plane of each sCMOS pixel could be calculated as 65 nm in both *x*- and *y*-direction. The image size of the hyperspectral image was 100 mm × 100 mm.

Two-photon imaging

With two-photon excitation, there are usually spectral overlap between second harmonic generation (SHG) and lignin fluorescence signals. To obtain SHG images of the spruce samples, a two-photon hyperspectral imaging system was employed [19]. A femto-second laser with central wavelength at 830 nm (Ti:sapphire laser, Avesta) was used as excitation source and the laser beam was focused onto the sample through an objective (Obj; UPLSAPO 60XW, Olympus). Via a scanning system, the laser beam was scanned along the *y*-direction. The signals arising from the line-shaped excitation area were then collected into a spectroscopic system. For each *y*-scanning, a y-λ image, including both spatial and spectral information, were recorded by a CMOS camera (ORCA-Flash4.0 V2, Hamamatsu). A x-y-λ HSI stack can be obtained by scanning the sample along the *x*-direction. After obtaining a HSI stack, linear unmixing method [20] was applied to deconvolve the stack into fluorescence and SHG images, based on two fluorescent and one SHG spectral bases.

Metal treatment of cellulose-based earphone diaphragms

The earphone driver unit (14.3 mm diameter) was purchased from Inventec (Taipei, Taiwan). Its cellulose-based diaphragm (10 mm diameter, 5.5 mg) is 80% cotton fiber blended with wool. Aluminum perchlorate nonahydrate (20.5 mM) or sodium perchlorate (61.5 mM) was dissolved in 10% acetic acid in methyl propylene glycol ether (v/v) and sonicated. The acidity prevented the formation of aluminum hydroxide precipitates. The metal stock solution (5 μL) was added onto the diaphragm and low-speed centrifugation (200 ppm, 1 min) was applied to enhance liquid spreading. The diaphragm was oven dried for 30 min at 60 °C, and then vacuum dried at r.t.

Frequency response measurement

Before acoustic measurements, the earphone diaphragm was oven dried (60 °C) for 5 min and stabilized at r.t. for 5 min. The frequency response of the metal-treated diaphragm was measured using an EA-2 measurement system (IEA Electro-Acoustic Technology, Taipei, Taiwan) with CLIO 12.0 signal analysis software (Audiomatica, Firenze, Italy). Sound pressure level was measured using an ear simulator kit with a 1/4 inch measurement microphone (GRAS, Holte, Denmark), which has a flat frequency response from 10 Hz-21 kHz. This setup is designed specifically for measuring the acoustic output of small-diameter earphone drivers.

Young's modulus measurement

Yomoshi paper (85% cotton fiber blended with wool) was purchased from Takeo (Tokyo, Japan) and cut into 5 x 1 cm strips (0.39 mm thickness, 240 mg). Metal stock solutions (220 µL) were added evenly over the strip, followed by oven drying for 30 min at 60 ºC and then vacuum drying. Young's modulus was measured using the material parameter measurement module (Klippel R&D, Dresden, Germany), using a modified vibration beam technique originally based on ASTM E 756-93 (Standard test method for measuring vibration-damping properties of materials) [21].

Statistical analysis

Elemental profiles of spruce and maple samples were compared using principal component analysis to visualize similarities. The elements included in the computations included Al, B, Ba, Ca, Cu, Fe, K, Mg, Mn, Na, P, and Zn. Principal component analysis was computed using Origin 2016 software (OriginLab, Northampton, MA) by choosing the correlation matrix. Welch's t-test was calculated using Microsoft Excel software. To visualize dissimilarities, multidimensional scaling was computed using PAST software version 3.23 [22], by choosing principal coordinate analysis with Gower similarity matrix with the exponent of 2.

II. Tables

Table S1. Wood samples from antique instruments

Instrument	Assigned origin	Spruce sample # and location	Maple sample # and location	Sample source
viola, c. 1619	Nicolo Amati	SC1 (top plate)	MC1 (back)	John Harte
cello, c. 1701	Antonio Stradivari	SC2 (top plate)		Guy Rabut
cello, c. 1707	Antonio Stradivari		MC2	Guy Rabut
violin, c. 1709	Antonio Stradivari	SC3 (top plate)		Guy Rabut
violin, c. 1717	Antonio Stradivari		MC3 (back, near lower edge)	Rene Morel [23]
cello, c. 1720	Antonio Stradivari	SC4 (top plate)		Guy Rabut
violin, c. 1725	Antonio Stradivari		MC4 (original neck, heel region)	Chimei Museum [24]
violin, c. 1730	Antonio Stradivari	SC5 (top plate)		Guy Rabut
cello, c. 1731	Antonio Stradivari		MC5 (back)	Rene Morel [23]
violin, c. 1740	Guarneri del Gesù	SC6 (top plate)		Guy Rabut
violin, c. 1741	Guarneri del Gesù		MC6 (back, center region)	Rene Morel [23]
quinton-violin, c. 1750	French	SA1 (top, chin-rest area)	MA1 (back, shoulder-rest area)	Renner
violin, c. 1780	French	SA2 (top, chin-rest area)	MA2 (back, shoulder-rest area)	Renner
violin, c. 1790	English	SA3 (top, chin-rest area)	MA3 (back, shoulder-rest area)	Renner
neck heel, c. 1800	Italian		MA4 (heel extension repair on MC4)	Chimei Museum [24]
violin, c. 1850	German	SA4 (top, chin-rest area)	MA5 (back, shoulder-rest area)	Renner

Table S2. Wood samples from old and new tonewood planks

Wood Sample	Assigned origin	Spruce sample #	Maple sample #	Sample source
Modern spruce #1	European tonewood	SM1		Chiao
Modern spruce #2	European tonewood	SM2		Chiao
Modern spruce #3	European tonewood	SM3		Chiao
Modern spruce #4	European tonewood	SM4		Lee
Modern spruce #5	European tonewood	SM5		Lee
Old building spruce #1	European, c. 1704*	SO1		Goldsmith
Old building spruce #2	European, c. 1708*	SO2		Goldsmith
Old building spruce #3	European, c. 1719*	SO3		Goldsmith
Modern maple #1	European tonewood		MM1	Chiao
Modern maple #2	European tonewood		MM2	Chiao
Modern maple #3	European tonewood		MM3	Chiao
Modern maple #4	European tonewood		MM4	Lee
Modern maple #5	European tonewood		MM5	Lee
Modern maple #6	European tonewood		MM6	Lee
Modern maple #7	European tonewood		MM7	Lee

* Dendrochronology dating of outer rings

Table S3. Characterization of wood from an antique Chinese zither (c. 9-10th century)

wood origin	species identification	^{14}C counts	^{14}C age (year BP)	error (±)	median calendar year	calendar year (2 sigma) / probability
top plate	Chinese fir *Cunninghamia lanceolata*	17494	1352	61	674	564-777 (0.989)<br>793-802 (0.005)<br>845-855 (0.006)
back plate	Chinese fir *Cunninghamia lanceolata*	55498	1213	34	811	690-750 (0.182)<br>761-892 (0.818)

Note: This antique zither is thought to originated from the Tang Dynasty (618-907 AD), based on its shape, building technique, and accessories. Radiocarbon dating results are consistent with the Tang Dynasty classification.

Table S4. X-ray diffraction data of modern and historical maples

	(200)				(004)			
	2θ	FWHM	domain size (nm)	d (nm)	2θ	FWHM	domain size (nm)	d (nm)
Modern SM1	14.935	1.702	3.297	0.397	23.022	0.184	30.864	0.259
Modern SM5	15.055	1.757	3.194	0.394	23.077	0.156	36.314	0.258
Building SO1	15.018	1.766	3.178	0.395	23.013	0.306	18.558	0.259
Building SO2	15.018	1.766	3.178	0.395	23.031	0.156	36.311	0.259
Building SO3	15.009	1.840	3.050	0.396	23.050	0.561	10.120	0.259
Amati SC1	15.202	1.757	3.195	0.391	23.197	0.156	36.321	0.257
Stradivari SC2	15.046	1.619	3.467	0.395	23.022	0.156	36.310	0.259
Stradivari SC3	14.917	1.720	3.262	0.398	23.004	0.147	38.578	0.259
Stradivari SC4	15.018	1.684	3.334	0.395	23.040	0.156	36.311	0.259
Stradivari SC5	15.009	1.610	3.486	0.396	23.050	0.138	41.153	0.259
Guarneri SC6	15.073	1.711	3.280	0.394	23.022	0.175	32.488	0.259

Note: FWHM = full width at half maximum

Table S5. The chemical composition of modern spruce and maple tonewood

	organic composition (% dry weight)*			
mean	cellulose	lignin	acetyl	hemicellulose saccharides
spruce SM1	45.5	32.8	1.8	19.9
spruce SM5	44.4	34.2	2.0	19.4
maple MM1	41.6	31.5	5.3	21.6
maple MM3	41.8	31.1	5.8	21.3
standard deviation				
spruce SM1	4.30	1.46	1.42	3.79
spruce SM5	2.93	0.93	2.25	1.64
maple MM1	2.51	1.37	1.27	1.68
maple MM3	2.49	0.79	0.76	2.24

* Average of three repeated experiments

Table S6. ICP-MS elemental analysis of antique violin and Cremonese spruces

	SA1	SA2	SA3	SA4	SC1	SC2	SC3	SC4	SC5	SC6
Al	5	4	1	2	14	5	0	25	9	1196
As	1	2	1	1	ND	ND	ND	ND	ND	ND
B	10	19	23	23	64	10	28	69	16	40
Ba	6	5	12	7	21	6	17	28	21	20
Ca	1279	1296	966	949	776	626	1759	1416	1586	6165
Cd	0	0	ND	0		0	1		1	0
Cl	1847	1847	1677	1171	3278	137	1666	7189	743	1325
Cr	1	1	0	0	0	1	1	1	1	4
Cu	6	9	1	4	15	1	6	20	4	6
Fe	13	20	5	3	40	50	38	90	54	143
Ge										
Hg	2	2	7	2	0	ND	ND	ND	1	0
K	2816	4289	984	1968	1449	370	1307	4124	935	1039
Li										
Mg	174	170	114	153	165	63	97	194	188	817
Mn	21	27	168	205	98	62	86	68	30	110
Na	3330	3491	1856	1895	1736	194	966	12744	831	1518
Ni	ND	ND	ND	1	0	ND	1	1	ND	1
P	118	79	40	56	227	90	447	264	148	426
Pb	19	39	2	5	1	ND	ND	18	1	ND
S	146	284	102	155	289	146	740	349	275	1030
Sb	ND	ND	ND	ND	ND	ND	ND	0	ND	ND
Se	ND	ND	ND	ND	ND	ND	ND	ND	ND	ND
Si	533	782	387	557	146	554	2312	299	864	4093
Sn	ND	ND	ND	ND	ND	ND	ND	2	ND	ND
Sr	5	7	6	3	9	3	3	6	25	14
Ti	ND	ND	ND	ND	1	ND	0	7	ND	34
Zn	45	46	5	93	7	4	39	77	12	8
Zr	1	1	ND	1	ND	4	3	ND	18	6
Analytical batch	4	4	4	4	2	4	4	2	4	4

Notes: Blank = not measured; ND = not detected; Concentrations rounded to 1 ppm

Table S7. ICP-MS elemental analysis of modern and old building spruces

	SM1	SM3	SM4	SM5	SO1	SO2	SO3	mean	SEM
Al	3	3	4	4	ND	2	9	3.5	1.1
As	ND	ND	ND	ND	ND	ND	ND	~0	
B	4	6	2	4	3	3	3	3.7	0.5
Ba	18	15	8	7	5	14	8	10.8	1.9
Ca	1408	1246	2019	1466	309	344	1135	1133	233
Cd	ND								
Cl	192	1051	1194	1537	1875	2126	1724	1386	244
Cr	ND	ND	2	ND	ND	ND	ND	<1	
Cu	1	0	1	ND	ND	ND	0	<1	
Fe	9	28	11	15	6	4	5	11.1	3.2
Ge									
Hg	ND	ND	ND	ND	ND	ND	ND	~0	
K	284	294	312	307	389	396	581	366	40
Li									
Mg	64	55	105	111	147	92	99	96	12
Mn	58	52	26	28	17	63	86	47	9
Na	32	20	4	18	18	43	134	38	17
Ni	ND	0	ND	ND	0	0	0	<1	
P	11	163	196	188	168	135	228	156	27
Pb	1	ND	0	ND	ND	1	ND	<1	
S	119	100	206	104	104	117	305	151	29
Sb	ND	0	ND	0	ND	ND	ND	<1	
Se	ND	ND	ND	ND	ND	ND	3	<1	
Si	515	122	233	129	140	171	145	208	53
Sn	ND	1	ND	0	ND	ND	ND	<1	
Sr	4	4	3	2	3	5	4	3.6	0.3
Ti	ND	3	ND	0	ND	0	0	<1	
Zn	13	11	15	12	ND	3	10	9.2	2.1
Zr	ND	ND	ND	ND	ND	ND	ND	~0	
Analytical batch	4	2	3	2	2	2	2		

Notes: Blank = not measured; ND = not detected; Concentrations rounded to 1 ppm. SEM = standard error of mean.

Table S8. ICP-MS elemental analysis of Cremonese maples

	MC1	MC1	MC3	MC3	MC4-left	MC4-right	MC4-right	MC5	MC6
Al	18	23	45	23	3	3	6	7	2931
As	ND	ND	ND	ND	ND	ND	1	ND	ND
B	22	43	46	54	18	11	12	42	34
Ba	8	15	5	4	1	1	2	1	9
Ca	2770	1085	255	758	1432	1881	3487	608	3339
Cd									
Cl		2936		9436	14214		5009	3473	
Cr	1	2	2	0	ND	ND	0	ND	7
Cu	6	2	64	116	5	6	13	4	52
Fe	95	43	135	104	12*	248*	60*	18	115
Ge	ND		ND			ND			ND
Hg	ND	ND	ND	1	2	ND	1	0	ND
K	1006	2472	4226	5001	4811	4306	5674	1441	565
Li	ND		ND			ND			ND
Mg	265	311	316	377	156	109	255	389	82
Mn	7	10	12	9	3	4	4	5	6
Na	470	772	4348	11316	3077	3067	3764	229	611
Ni	ND	0	21	18	0	ND	0	0	2
P	221	331	209	348	260	151	266	230	474
Pb	3	4	35	21	2	4	4	1	16
S		349		374	225		690	154	
Sb	ND	0	ND	0	ND	ND	ND	ND	ND
Se	ND	1	ND	ND	0	ND	ND	ND	ND
Si		246		217	251		274	234	
Sn	5	ND	3	0	ND	0	ND	ND	2
Sr	5	7	7	5	4	3	9	2	15
Ti	0	1	2	1	1	1	2	1	2
Zn	30	21	64	25	12	16	35	ND	120
Zr	ND	ND	0	ND	ND	ND	ND	ND	40
Analytical batch	1	2	1	2	2	1	3	2	1

Notes: Blank = not measured; ND = not detected; Concentrations rounded to 1 ppm;
* the neck heel region was originally fixed with nails by Stradivari [24]

Table S9. ICP-MS elemental analysis of modern maples

	MM1	MM2	MM2	MM3	MM4	MM5	MM6	MM6	MM7	MM7	mean	SEM
Al	2	6	1	3	2	2	3	10	5	3	3.7	0.85
As	ND	ND	ND	ND	ND	ND	ND	ND	ND	ND	~0	
B	4	2	3	2	5	3	3	4	5	8	3.9	0.61
Ba	1	0	0	0	2	1	2	3	3	4	1.6	0.40
Ca	533	1110	1523	1005	2248	2115	1005	1805	1181	1403	1393	170
Cd												
Cl	1241		935		1271	1138		1018		1489	1182	62
Cr	ND	ND	0	ND	ND	0	ND	ND	ND	ND	~0	
Cu	1	1	0	2	1	1	2	1	2	1	1.3	0.16
Fe	8	11	7	7	12	10	7	5	15	4	8.6	1.0
Ge		ND		ND			ND		ND		~0	
Hg	ND	ND	ND	ND	ND	ND	ND	ND	ND	ND	~0	
K	698	649	831	451	2360	1360	1058	1447	516	1272	1064	182
Li		ND		ND			ND		ND		~0	
Mg	395	414	486	317	493	306	284	361	291	427	377	25
Mn	4	2	2	1	4	3	9	12	13	16	6.7	1.7
Na	58	19	19	9	23	16	7	4	28	26	20.7	4.8
Ni	ND	ND	ND	ND	ND	ND	ND	0	ND	1	<1	
P	150	161	100	103	252	197	208	160	175	168	167	14
Pb	0	0	0	0	ND	0	0	ND	0	0	<1	
S	140		92		78	274		67		103	126	24
Sb	ND	ND	ND	ND	ND	ND	ND	ND	ND	0	~0	
Se	ND	ND	ND	ND	ND	ND	ND	ND	ND	ND	~0	
Si	216		104		261	190		103		129	167	21
Sn	ND	0	ND	0	ND	ND	0	ND	0	1	<1	
Sr	4	1	1	1	3	1	1	4	4	5	2.4	0.54
Ti	ND	0	ND	0	ND	ND	1	0	1	0	<1	
Zn	9	7	6	6	6	5	6	7	8	6	6.6	0.36
Zr	1	ND	0	ND	ND	ND	ND	ND	ND	1	<1	
Analytical batch	3	1	3	1	3	3	1	2	1	2		

Notes: Blank = not measured; ND = not detected; Concentrations rounded to 1 ppm. SEM = standard error of mean.

Table S10. ICP-MS elemental analysis of antique violin maples

	MA1	MA2	MA3	MA3	MA4	MA4	MA5
Al	6	3	270	3	4	1	1
As	ND	0	0	0	ND	ND	ND
B	9	8	21	22	8	8	9
Ba	10	33	3	3	1	2	19
Ca	845	1180	1131	1157	1609	2221	1140
Cd	0	ND	0	ND			0
Cl	389	985	979	1407		1709	1019
Cr	0	ND	0	ND	ND	0	0
Cu	2	1	2	0	4	2	1
Fe	13	11	12	7	18	16	8
Ge					ND		
Hg	ND	ND	ND	ND	ND	0	ND
K	1395	1872	667	1428	707	816	1474
Li					ND		
Mg	421	281	774	756	476	470	264
Mn	45	11	17	15	2	1	15
Na	1004	745	497	668	133	204	958
Ni	ND	0	1	1	ND	ND	0
P	157	147	164	321	248	217	102
Pb	1	1	1	1	2	3	1
S	196	194	105	234		563	103
Sb	ND	ND	ND	ND	ND	ND	ND
Se	ND	0	ND	ND	ND	ND	ND
Si	688	537	423	644		309	365
Sn	ND	ND	ND	ND	0	ND	ND
Sr	4	6	5	5	7	4	4
Ti	ND	ND	ND	0	1	ND	ND
Zn	3	3	5	3	42	8	3
Zr	2	1	1	4	ND	ND	1
Analytical batch	4	4	4	5	1	3	4

Notes: Blank = not measured; ND = not detected; Concentrations rounded to 1 ppm

Table S11. ICP-MS elemental analysis of chemically treated spruces

Element	SM5 + 0.1% oak ash	SM5 + $Ca(OH)_2$ pH 11	SM5 + 5% NaCl	SM5 soaked in water	SM5 + 2% K_2CO_3	SM5 + KOH pH 11.5	Oak ash	Oak ash soluble part
Al	39	1	ND	0	4	2	11484	365
As	ND	ND	ND	ND	ND	ND	1	ND
B	6	5	35	1	2	1	215	168
Ba	22	3	4	8	6	8	563	39
Ca	3307	5502	961	421	230	848	82688	8823
Cd	0	ND	ND	ND	ND	ND		0
Cl	225	56	242	281	429	360	189	606
Cr	1	0	0	0	0	ND	3	5
Cu	2	1	1	0	3	1	94	11
Fe	39	30	9	63	43	8	3186	7
Ge								
Hg	ND	ND	ND	ND	ND	ND	ND	ND
K	336	51	24	162	15872	3565	108736	400170
Li								
Mg	275	86	3	81	5	86	28291	1884
Mn	29	17	0	23	21	24	4580	2
Na	49	24	1981	2	1	9	6009	20152
Ni	ND	ND	ND	2	0	ND	8	1
P	38	35	24	194	98	119	7824	536
Pb	1	0	2	ND	0	0	5	ND
S	19	203	22	91	89	168	783	289
Sb	ND	ND	ND	ND	ND	ND	ND	ND
Se	1	0	ND	ND	ND	ND	1	ND
Si	879	827	908	832	452	468	572	1172
Sn	ND	ND	ND	ND	ND	ND	ND	ND
Sr	20	5	1	2	1	2	964	72
Ti	0	0	ND	0	0	ND	59	0
Zn	13	9	ND	10	7	11	238	2
Zr	ND	ND	ND	1	1	ND	6	ND
Analytical batch	4	4	4	5	5	5	3	4

Notes: Blank = not measured; ND = not detected; Concentrations rounded to 1 ppm

Table S12. ICP-MS elemental analysis of chemically treated maples

Element	MM1 + 0.1 % oak ash solution	MM1 + 1% alum solution	MM1 + $Ca(OH)_2$ pH 11	MM1 + 5% NaCl	MM1 soaked in water	MM1 + 2% K_2CO_3	MM1 + KOH pH 11.5
Al	71	665	23	0	5	17	8
As	ND	ND	ND	ND	ND	ND	ND
B	9	5	7	122	2	1	3
Ba	20	1	1	3	1	4	1
Ca	3472	827	4015	789	903	541	1154
Cd	0	ND	ND	ND	ND	ND	ND
Cl	510	307	641	1489	509	568	506
Cr	1	1	1	ND	ND	ND	0
Cu	2	1	2	1	1	1	0
Fe	38	44	39	11	22	18	12
Ge							
Hg	ND	ND	ND	ND	ND	ND	ND
K	340	90	67	19	102	12839	2654
Li							
Mg	373	3	101	4	200	22	236
Mn	20	0	3	0	3	3	2
Na	58	22	68	4358	0	2	4
Ni	ND	ND	ND	ND	0	ND	ND
P	118	67	58	36	217	161	255
Pb	ND	1	1	1	0	0	ND
S	331	154	196	18	222	111	244
Sb	ND	ND	ND	ND	ND	ND	ND
Se	1	1	1	ND	ND	ND	ND
Si	1680	605	1139	777	1218	886	1176
Sn	ND	ND	ND	ND	ND	ND	ND
Sr	24	ND	19	1	3	2	3
Ti	0	4	2	ND	2	0	0
Zn	4	1	10	1	10	6	7
Zr	1	1	3	ND	2	1	ND
Analytical batch	4	4	4	4	5	5	5

Notes: Blank = not measured; ND = not detected; Concentrations rounded to 1 ppm

Table S13. ICP-MS analysis of NIST Standard Reference Material 1575a Pine Needles

	1575a	1575a	1575a	1575a	Datasheet reference value
Analytical batch	2	3	4	5	
Al	569	585	532	570	580
As	ND	ND	ND	0.038	0.039
B	10.8	9.0	9.6	9.0	9.6
Ba	5.3	5.5	6	6	6
Ca	2613	2411	2360	2557	2500
Cd			0.219	0.219	0.233
Cl	1264	1149	410	540	421
Cr	ND	0.3	0.4	0.5	0.5
Cu	3.1	3.1	2.8	2.8	2.8
Fe	37	42	44	46	46
Ge					
Hg	0.0379	0.0409	0.0366	ND	0.0399
K	4339	4276	4001	4190	4170
Li					
Mg	997	1071	1005	1005	1060
Mn	488	477	458	474	488
Na	59	59	64	62	63
Ni	1.32	1.36	1.55	1.39	1.47
P	945	1004	1026	1066	1070
Pb	0.113	0.149	0.173	0.169	0.167
S	146	143	69	63	
Sb	ND	ND	ND	ND	
Se	ND	ND	ND	ND	0.099
Si	688	650	536	685	
Sn	ND	ND	ND	ND	
Sr	7.0	6.7	6	6	
Ti	1.6	1.0	0.9	3.2	
Zn	37	34	36	39	38
Zr	ND	0.5	1	11	

Notes: Blank = not measured; ND = not detected; Concentrations rounded to 1 ppm

III. Figures

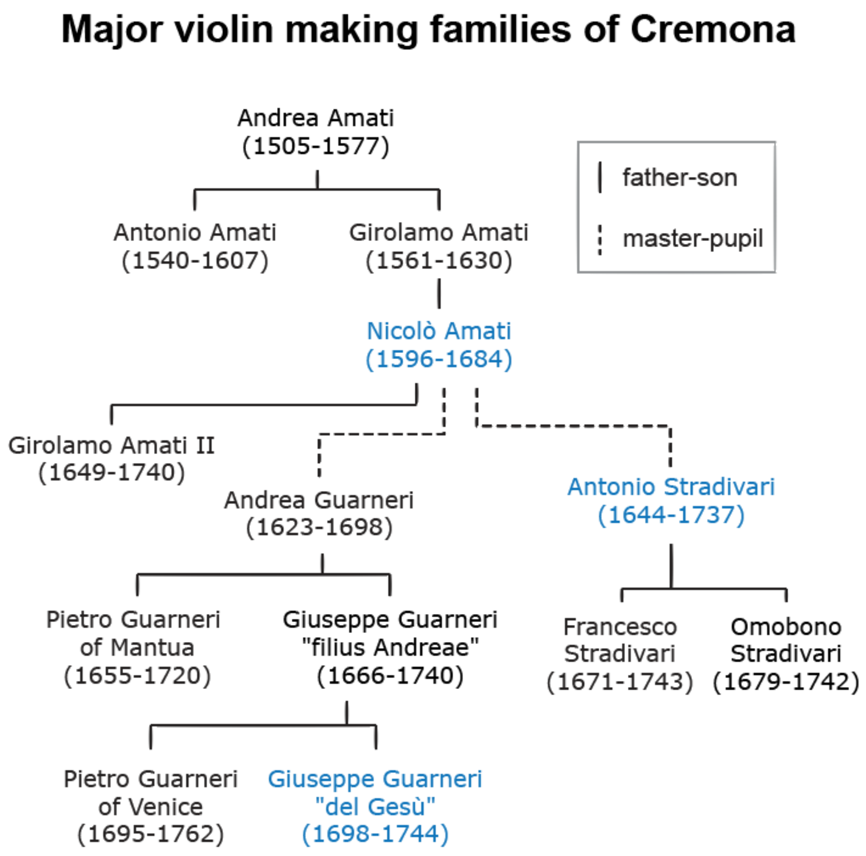

Figure S1. Three major violin making families during the Golden Age of Cremona (1550-1750). The maker names in blue are analyzed in this study. The modern violin was invented by Andrea Amati before 1550.

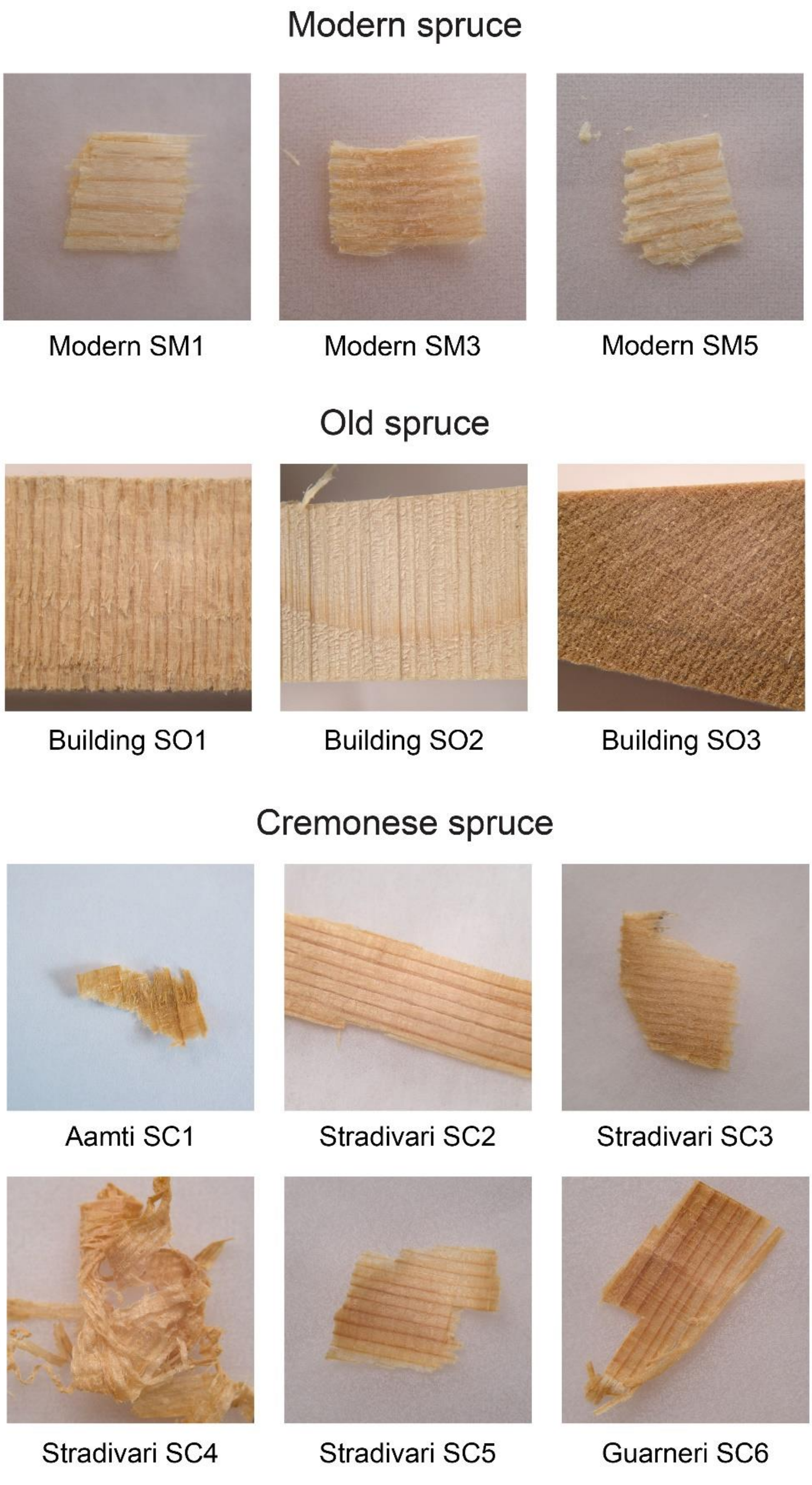

Figure S2. Photographs of Cremonese and control spruce samples. The Cremonese spruce scrapings, removed during repairs, are only slightly more yellow compared to modern controls. The spruces taken from old buildings appear to be well preserved and suitable as for instrument making.

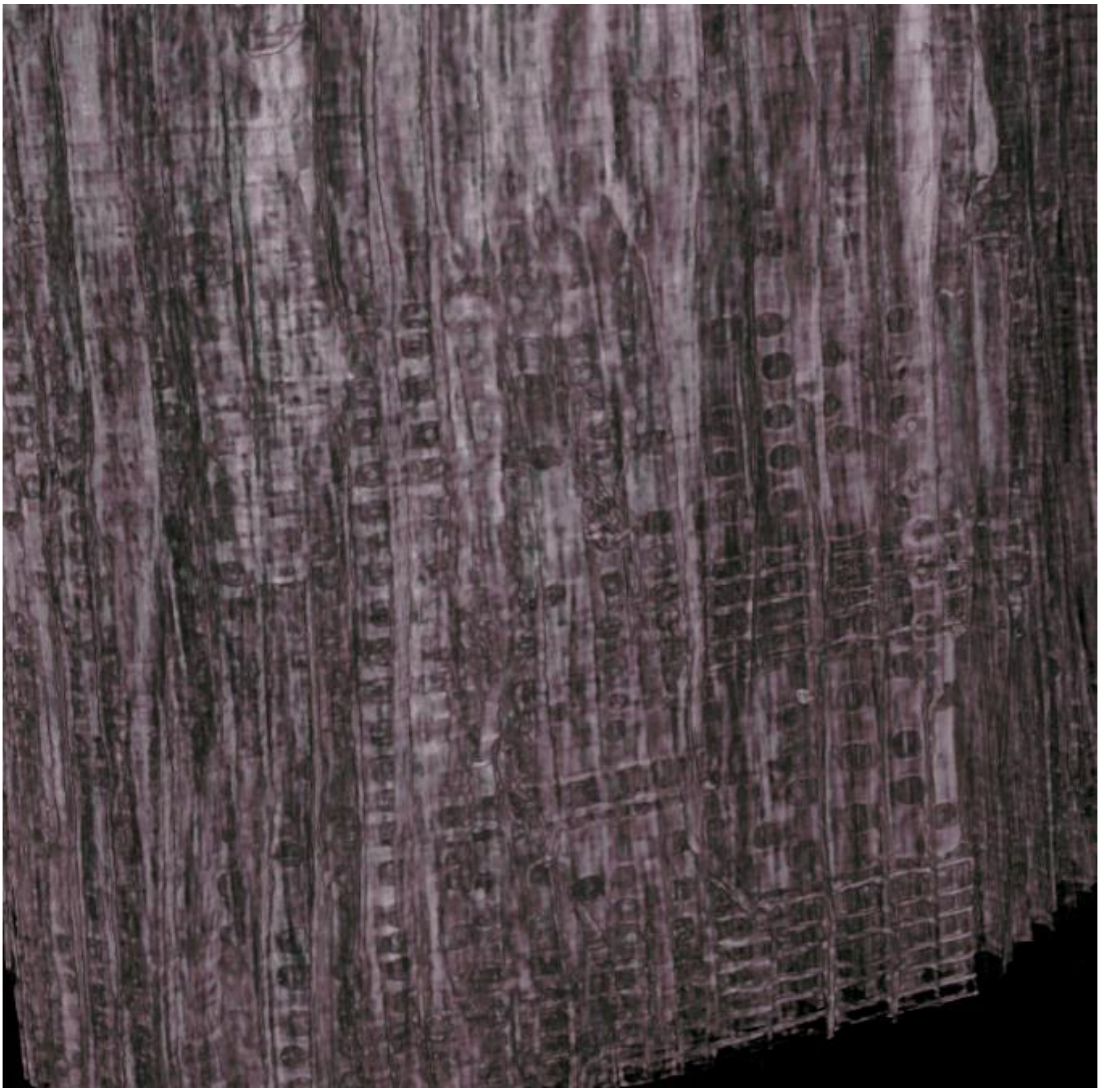

Figure S3. Volume rendering of X-ray tomography data for modern spruce (SM1). The image was rendered with 70% transparency in order to show the structure of the sample and the absence of defects and/or fungus. Colors were artificially generated.

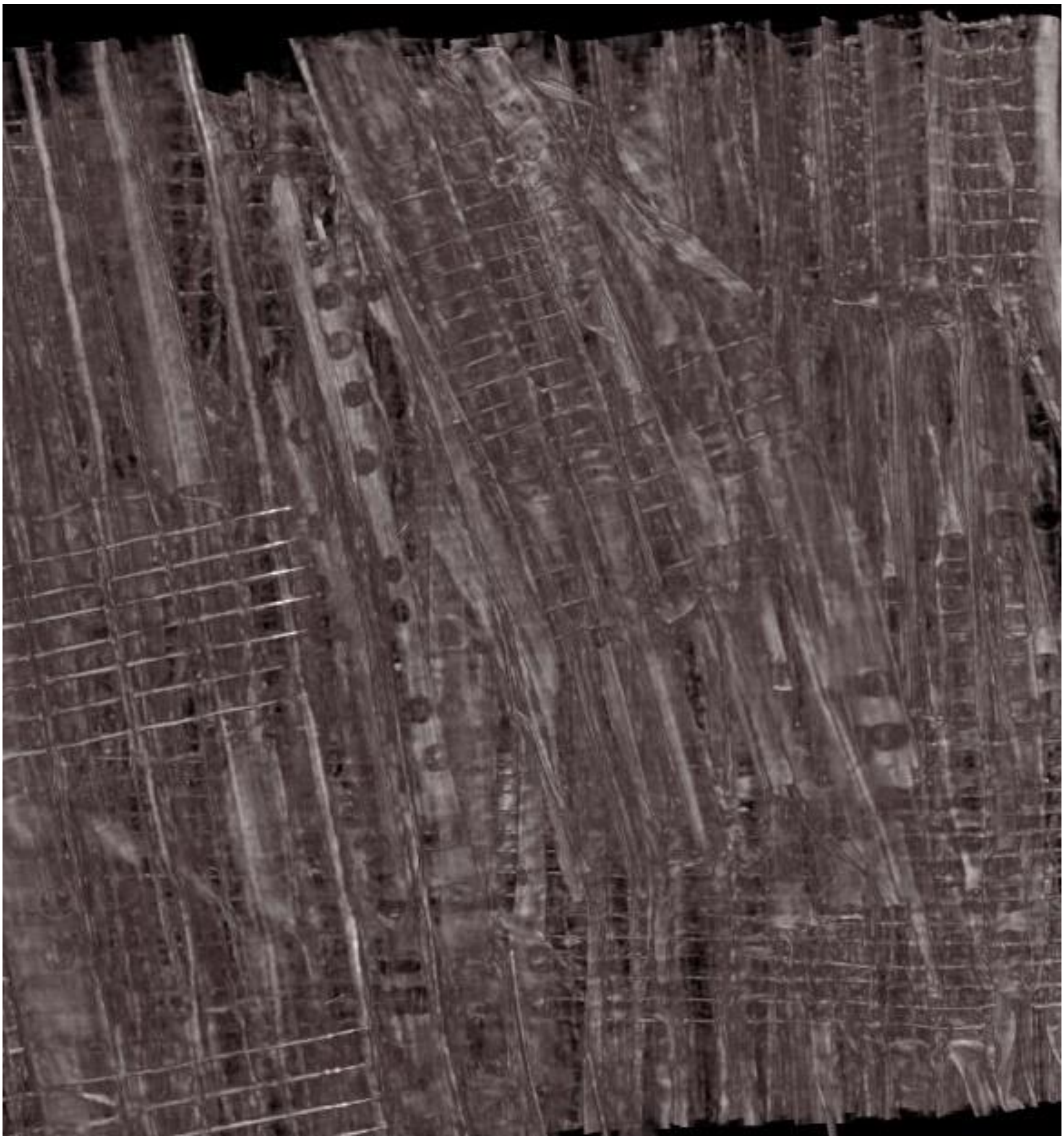

Figure S4. Volume rendering of X-ray tomography data for Amati spruce (SC1). The image was rendered with 70% transparency in order to show the structure of the sample and the absence of defects and/or fungus. Colors were artificially generated.

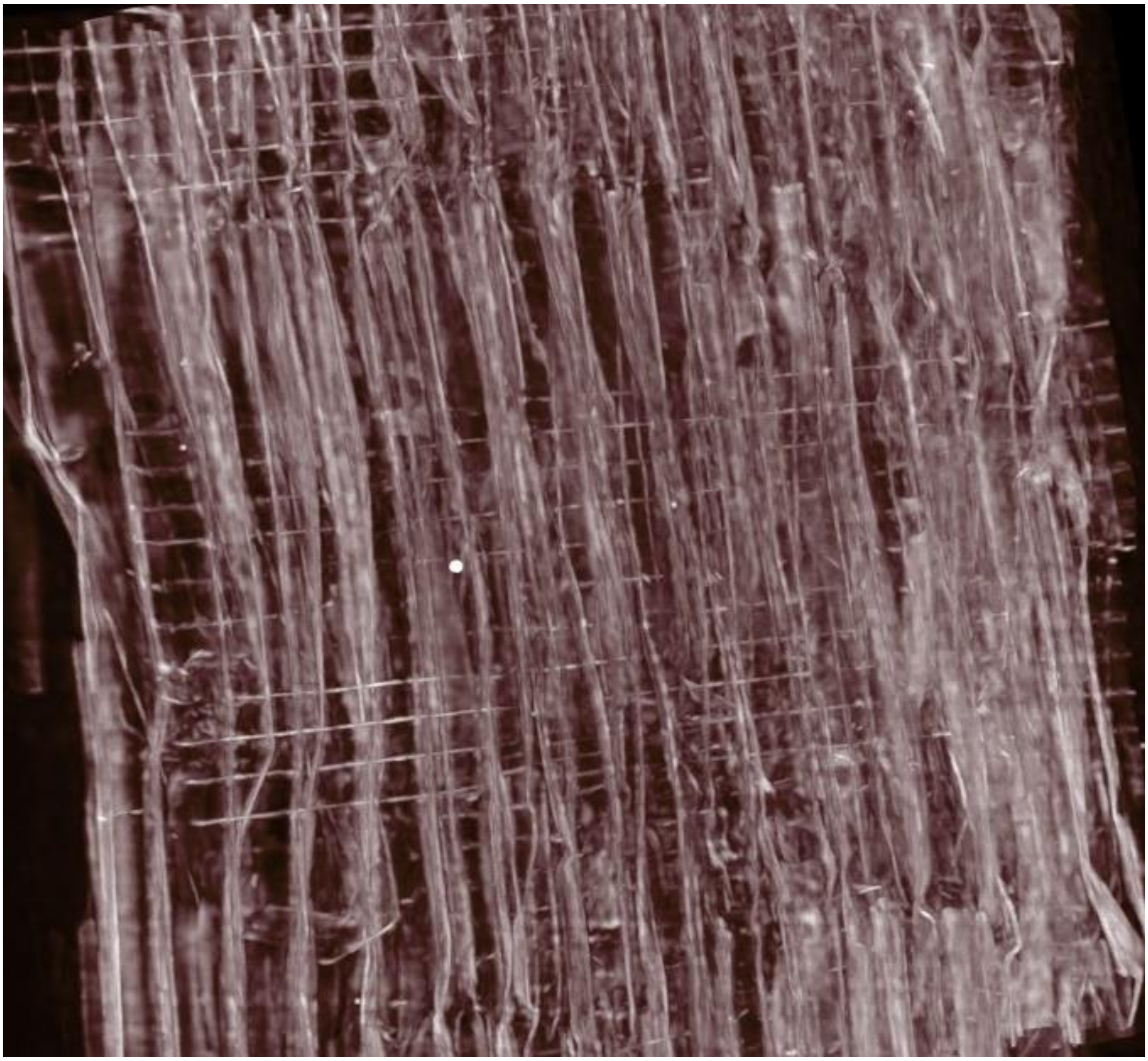

Figure S5. Volume rendering of X-ray tomography data for Stradivari spruce (SC4). The image was rendered with 70% transparency in order to show the structure of the sample and the absence of defects and/or fungus. Colors were artificially generated.

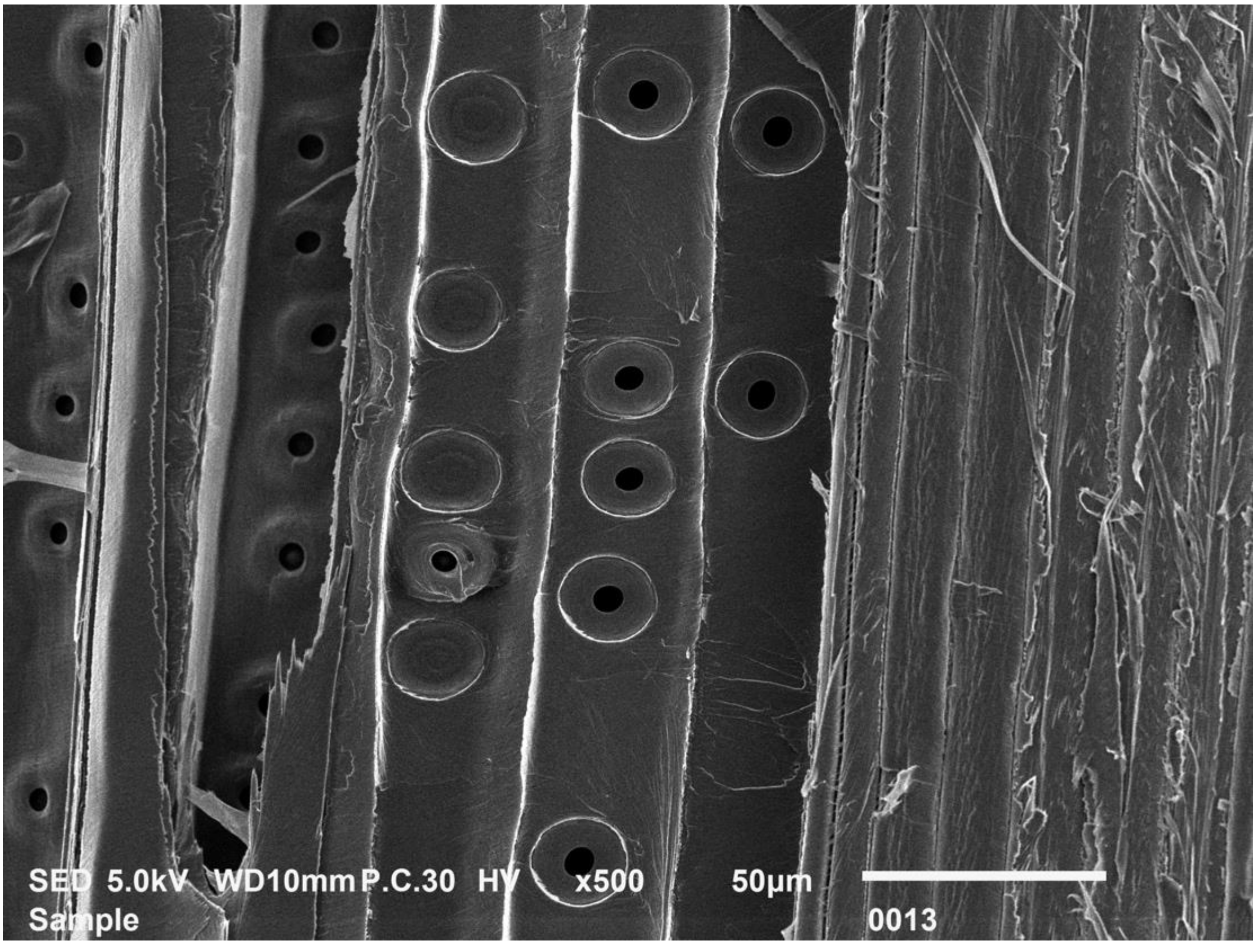

Figure S6. SEM image of modern spruce (SM1). The bordered pits of tracheid cells are clearly visible.

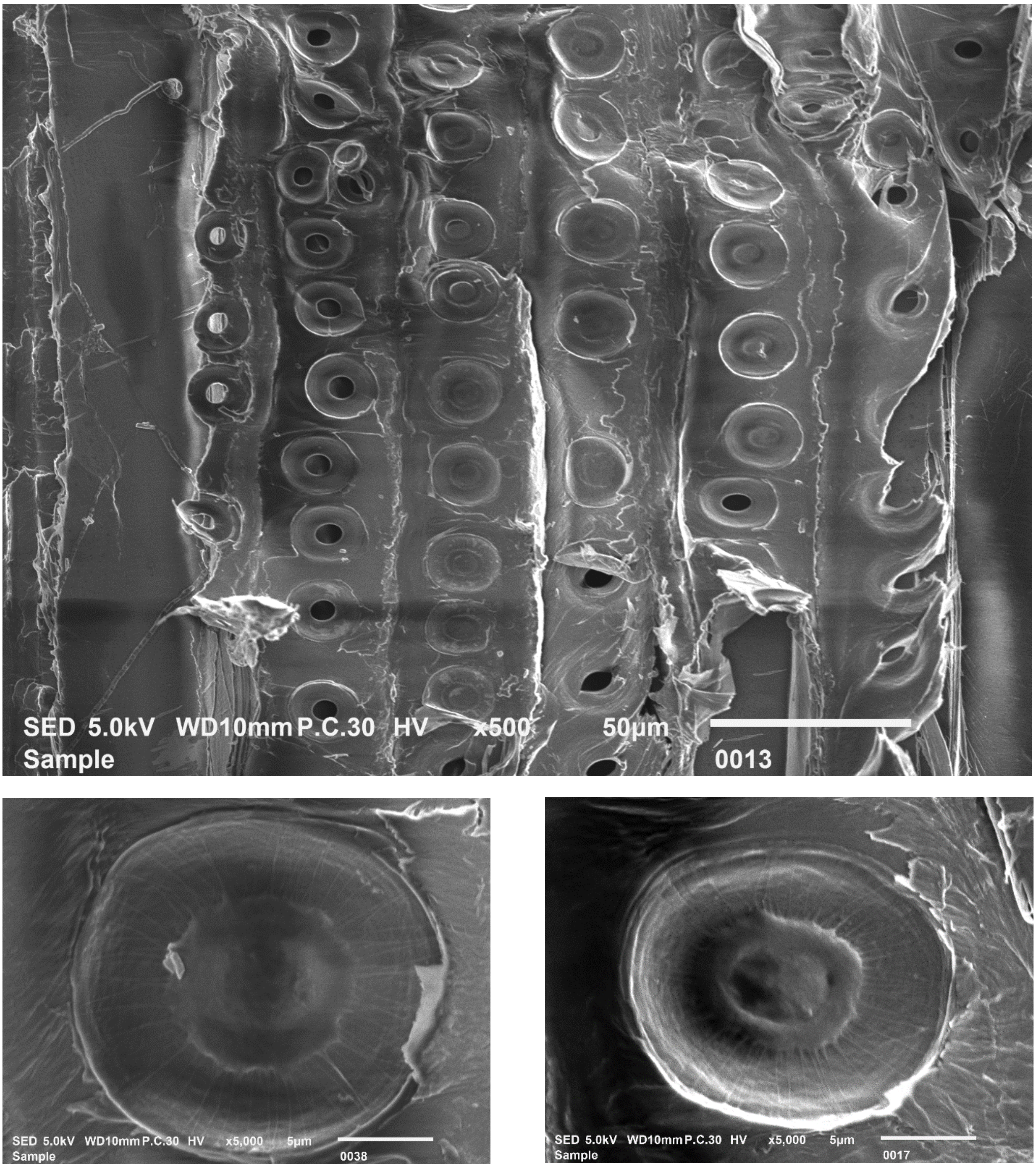

Figure S7. SEM images of Amati spruce (SC1). Shown below are torus/margo structures of intact pit membranes. No signs of partially degraded pit membranes are found.

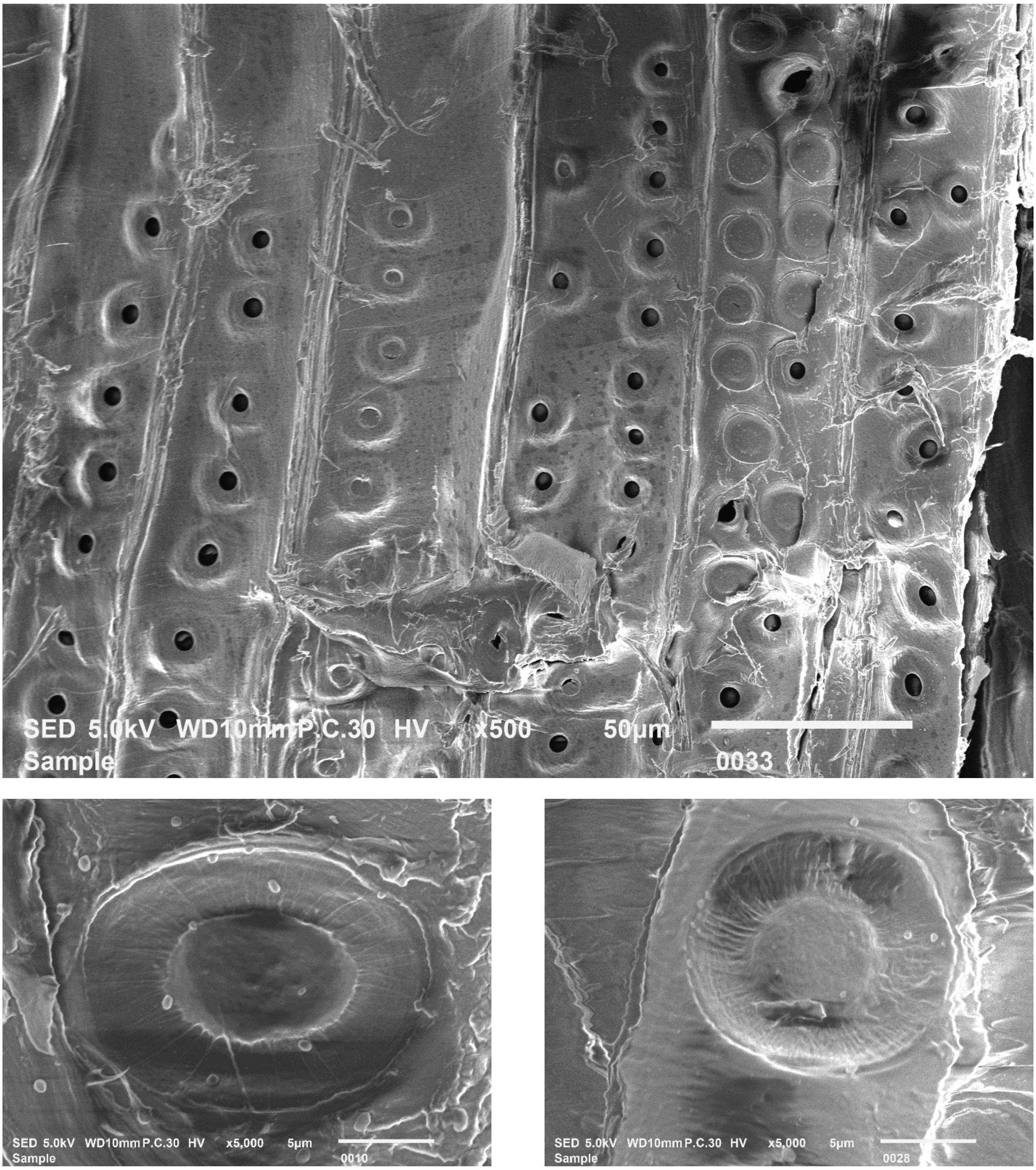

Figure S8. SEM images of Stradivari spruce (SC4). No signs of partially degraded pit membranes are found. Bacteria-like particles can be observed around some of the torus/margo structures of intact pit membranes (shown below). When or how the bacteria reached these locations cannot be ascertained.

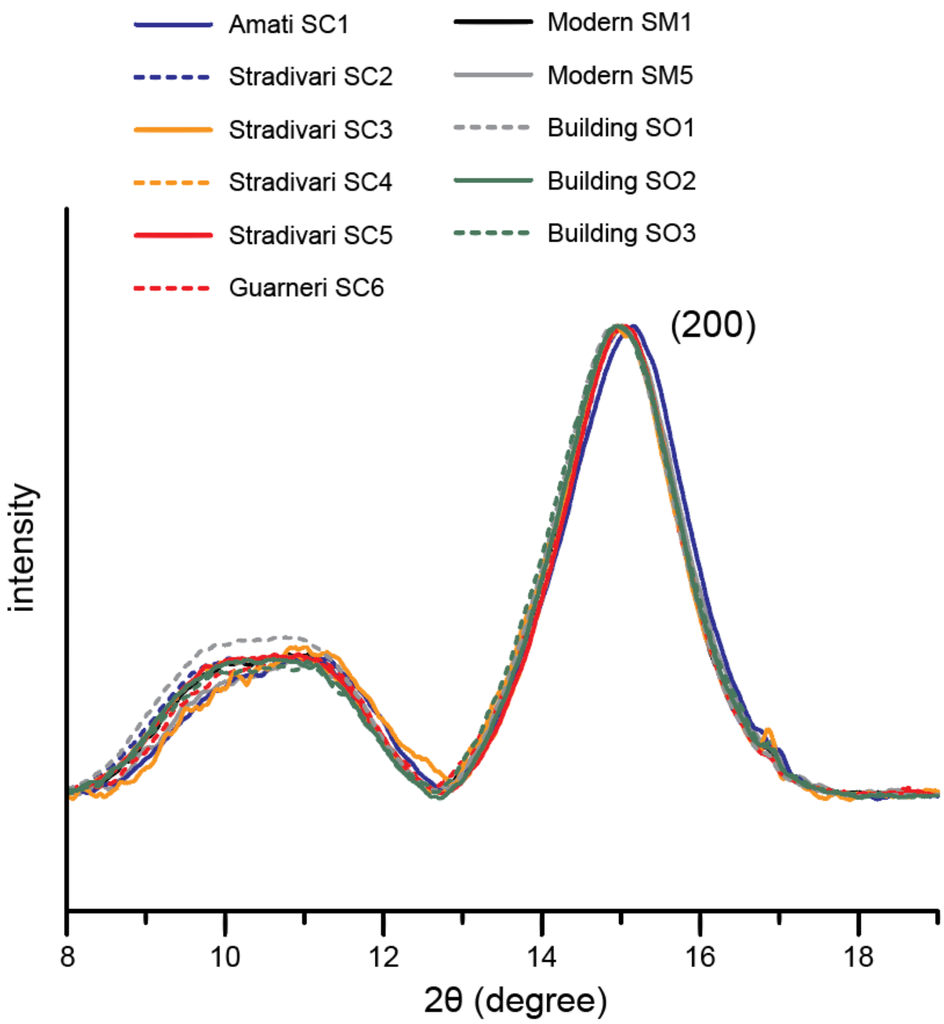

Figure S9. X-ray diffraction peaks of (200) for modern and historical spruce samples.

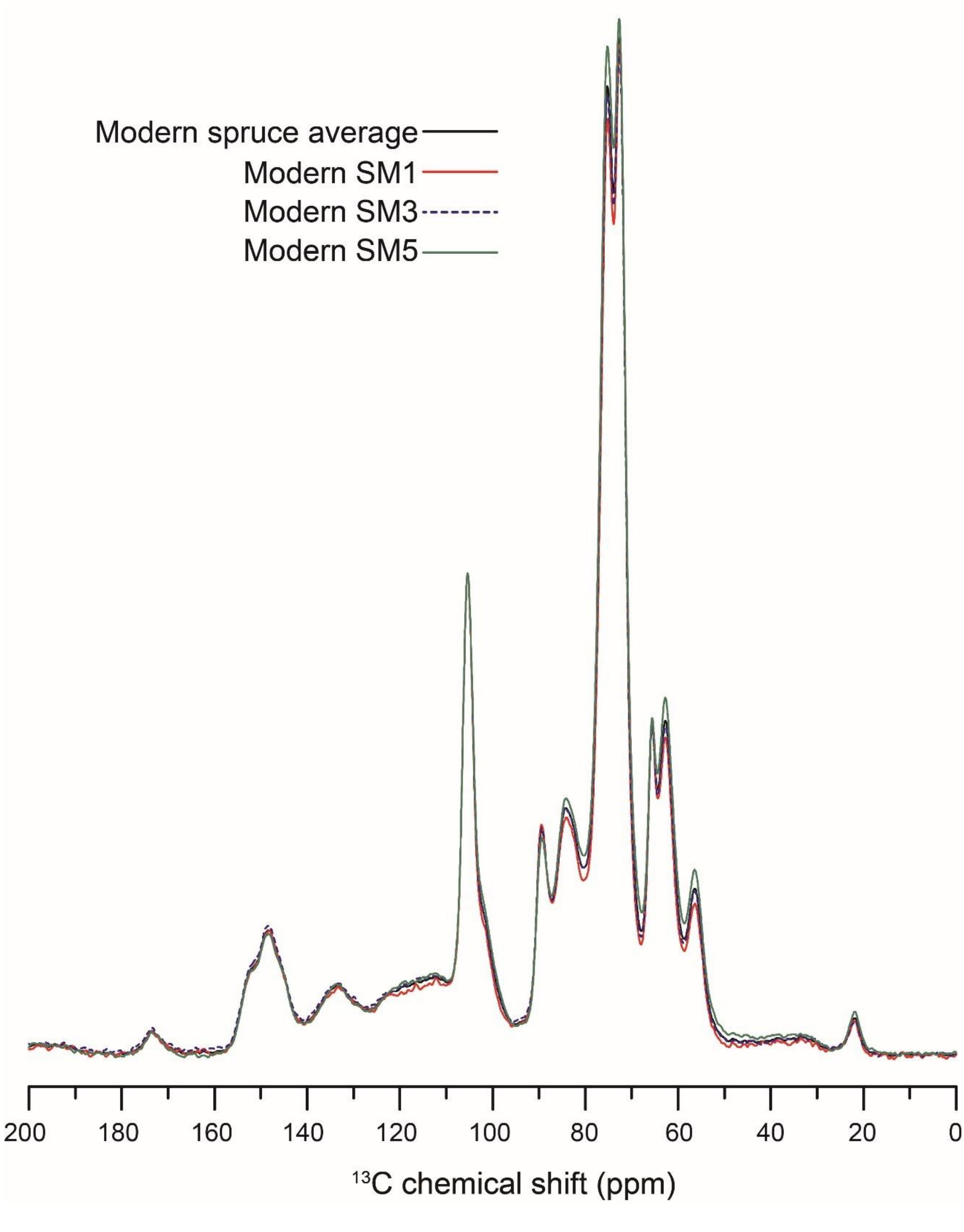

Figure S10. $^{13}C\{^{1}H\}$ multiCP NMR spectra of modern spruces and their averaged spectrum.

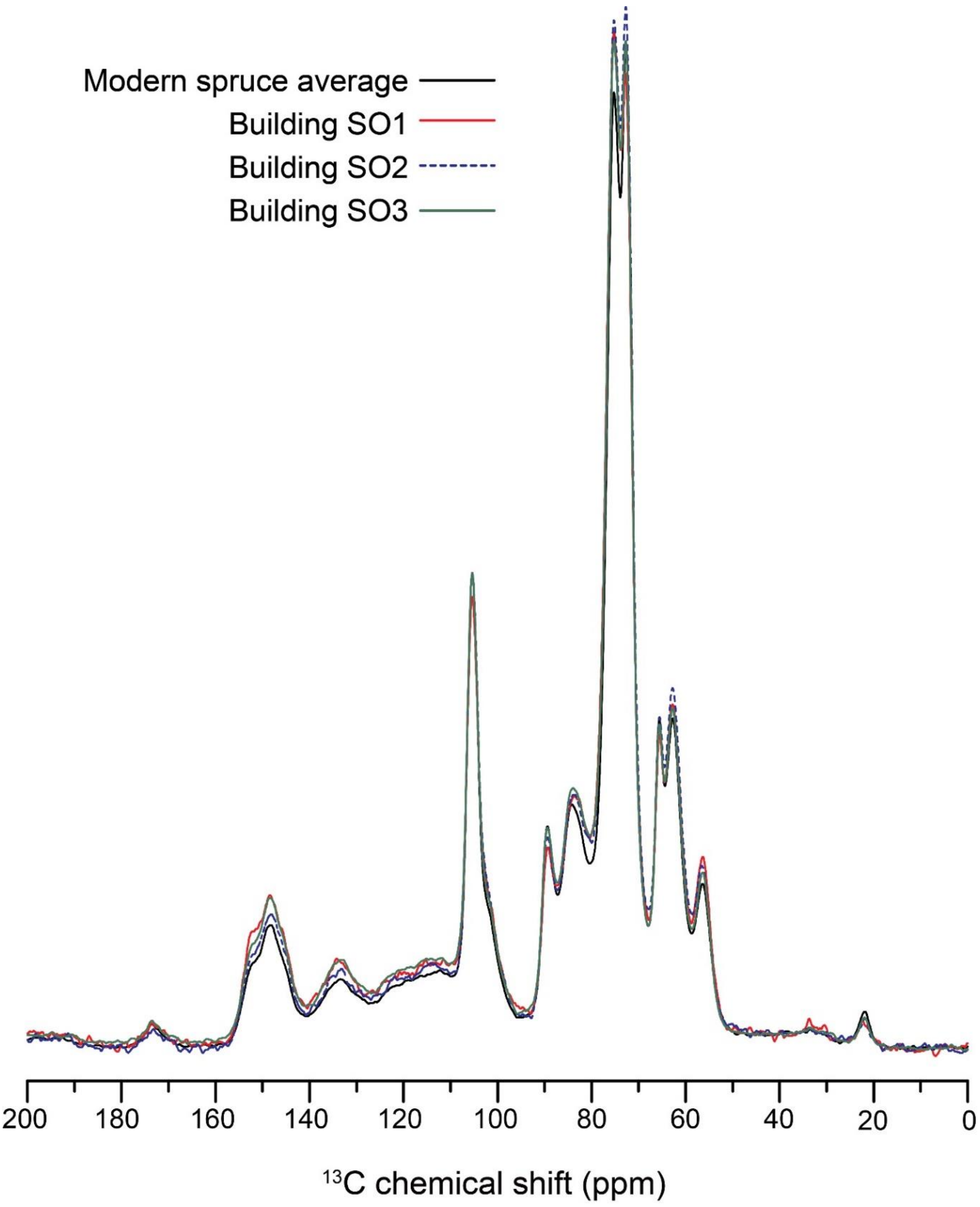

Figure S11. $^{13}C\{^1H\}$ multiCP NMR spectra of spruce specimens from old buildings.

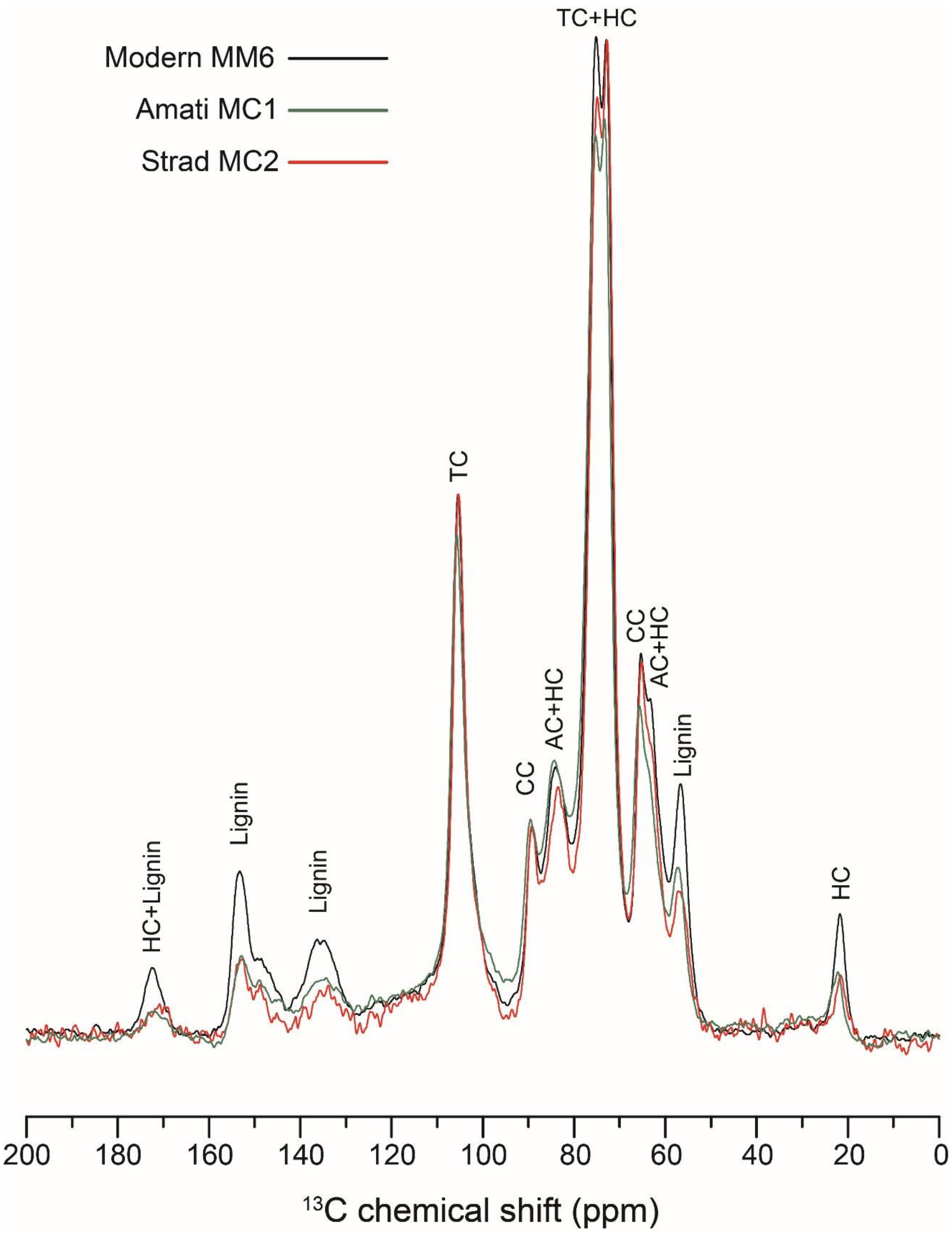

Figure S12. $^{13}C\{^{1}H\}$ multiCP NMR spectra of maple specimens: Amati viola (MC1), Stradivari cello (MC2), and modern tonewood (MM6). The peaks are assigned to total cellulose (TC), crystalline cellulose (CC), amorphous cellulose (AC), hemicellulose (HC), and lignin. Cremonese maples show signal decreases due to deacetylation (22, 173 ppm), demethoxylation (56 ppm), and hemicellulose decomposition (63 ppm, 75 ppm). Signal decreases in the aromatic region (125-160 ppm) may be due to lignin oxidation and peak broadening.

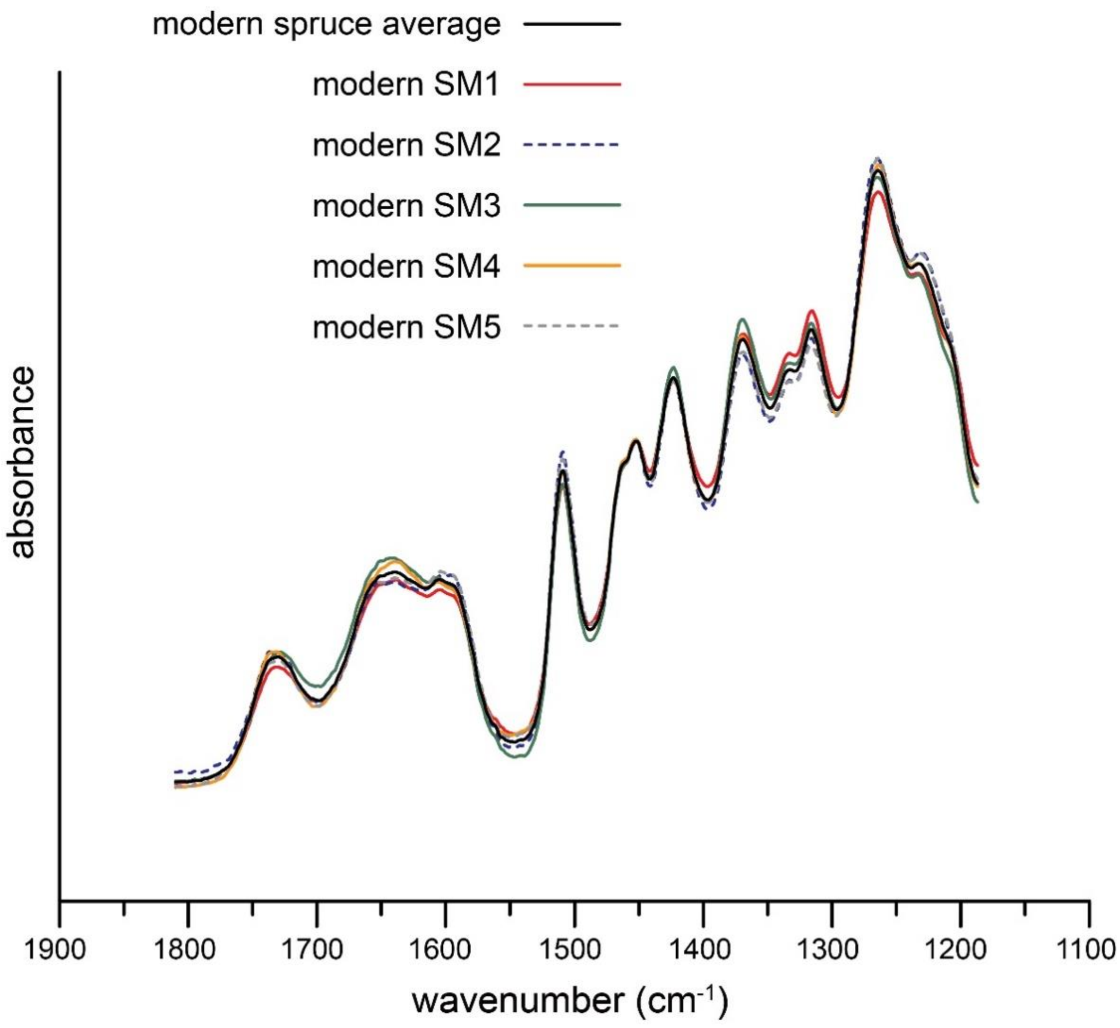

Figure S13. Infrared absorption spectra of modern spruce specimens and their averaged spectrum.

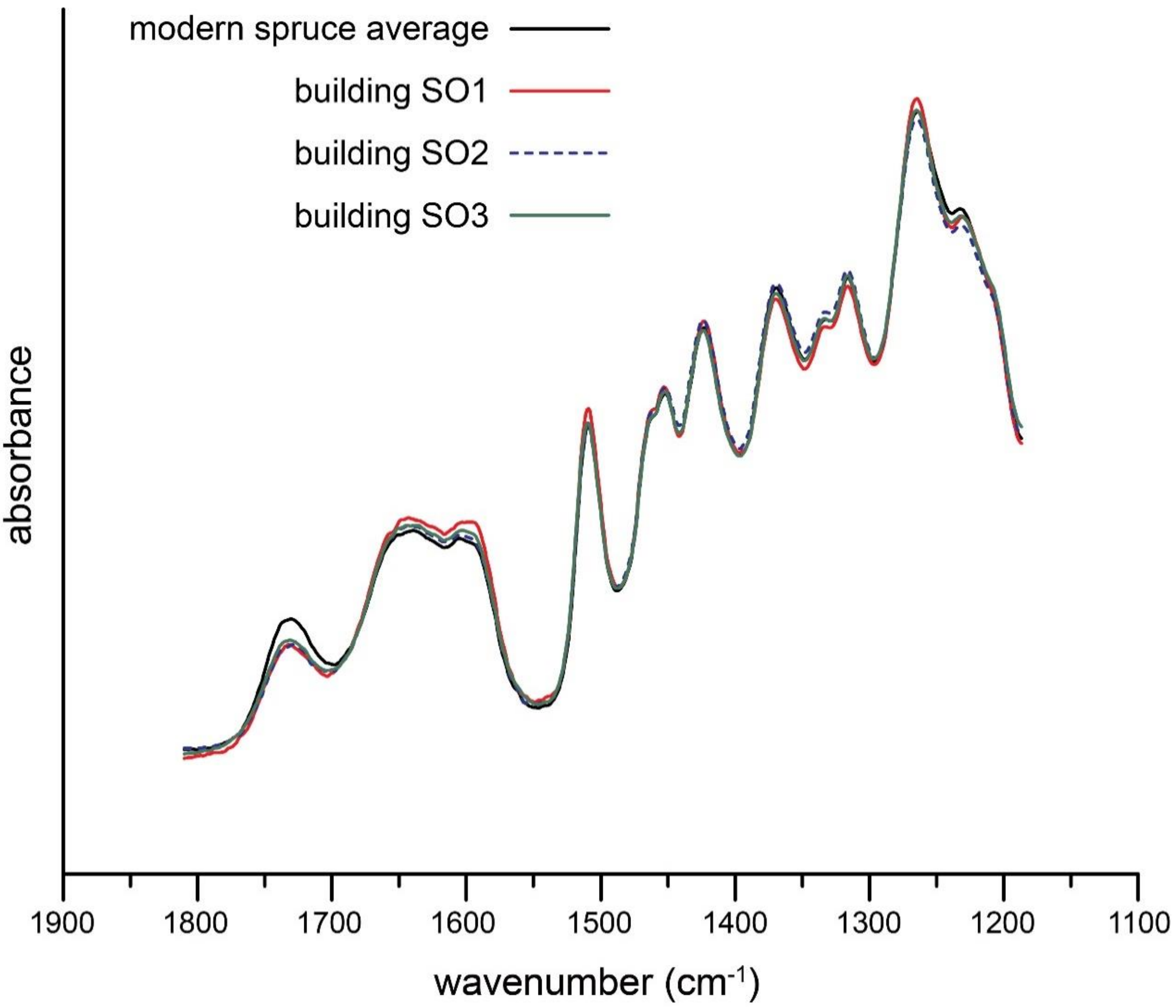

Figure S14. Infrared absorption spectra of spruce specimens taken from old buildings. A slight signal decrease in old building samples at 1740 cm^{-1} suggests partial deacetylation of hemicelluose.

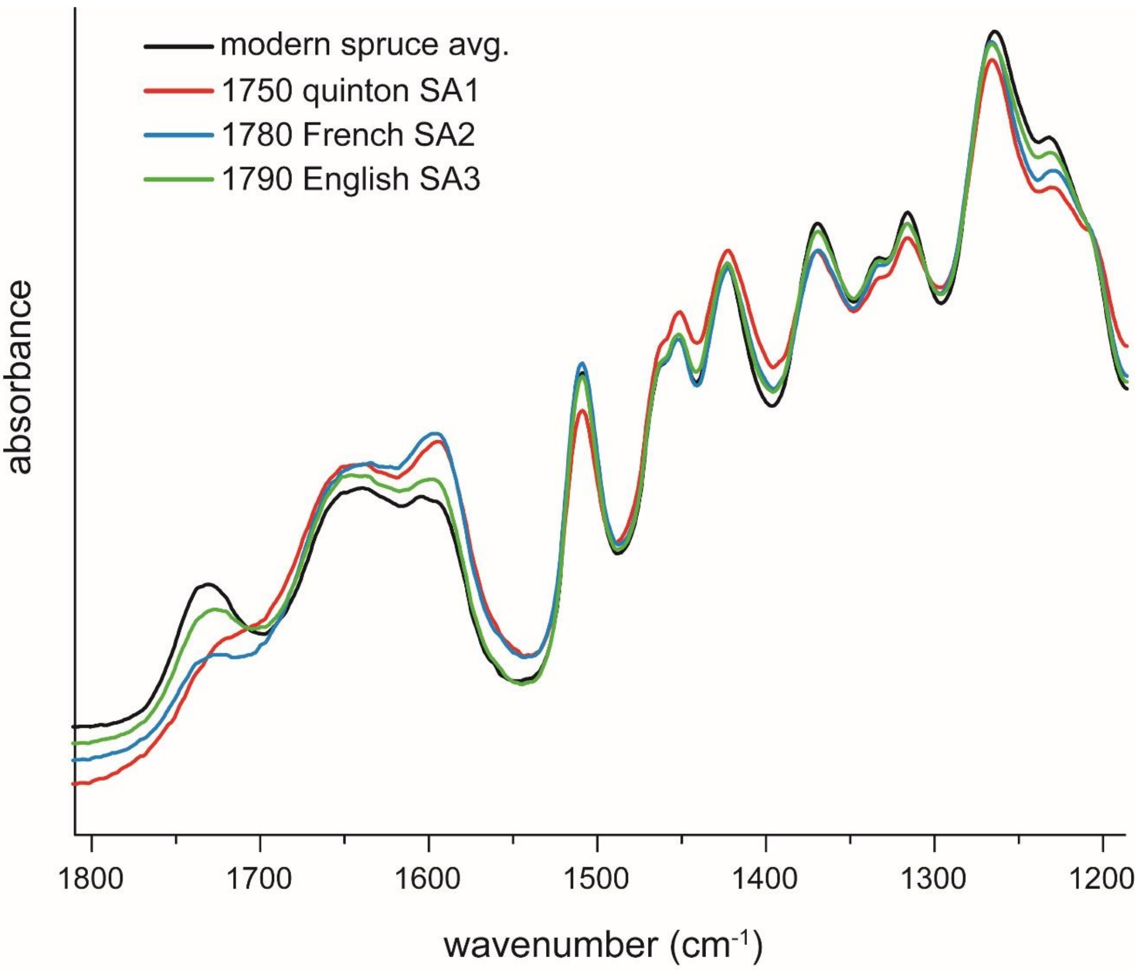

Figure S15. Infrared absorption spectra of spruces from unexceptional old European violins. The signal decrease at 1740 cm^{-1} suggests partial deacetylation of hemicelluose.

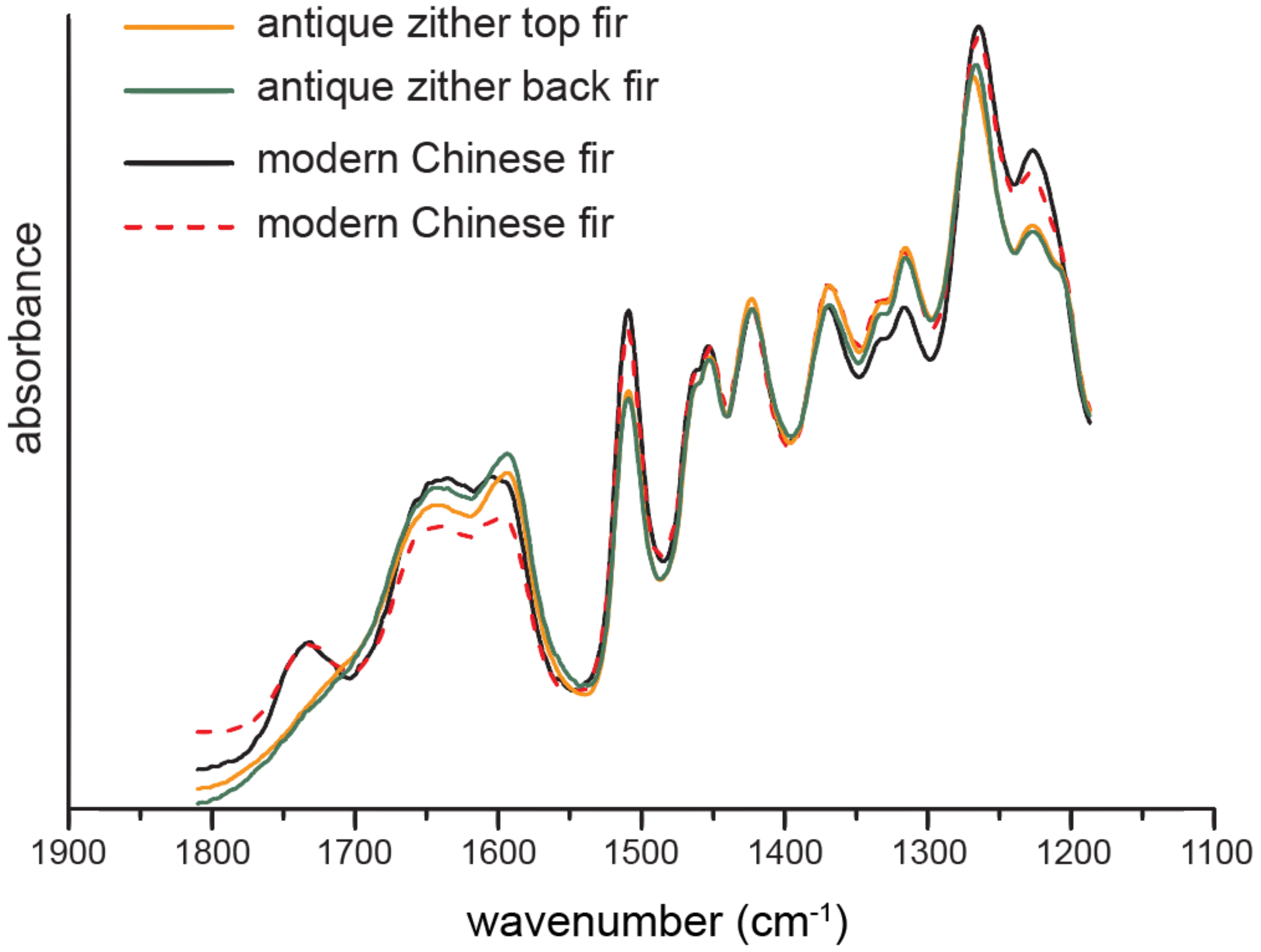

Figure S16. Infrared absorption spectra of modern Chinese fir and aged Chinese fir (>1000 years) from an antique Chinese guqin (7-string zither). The signal decrease at 1740 cm^{-1} suggests severe deacetylation of hemicelluose.

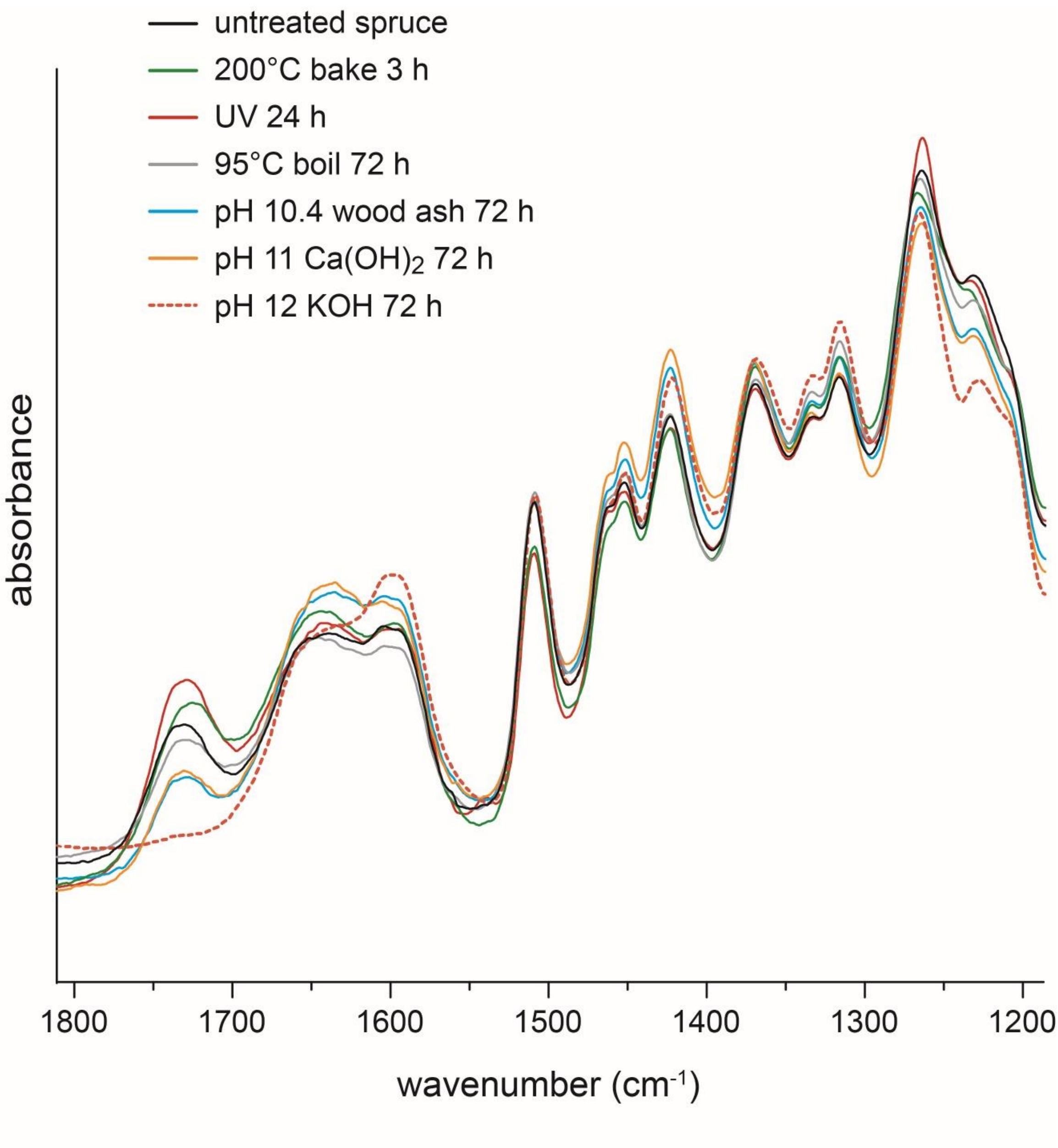

Figure S17. Infrared absorption spectra of modern spruce (SM5) after artificial treatments. Signal decrease at 1735 cm^{-1} and 1210 cm^{-1} represent the loss of acetyl groups and hemicellulose decomposition, respectively.

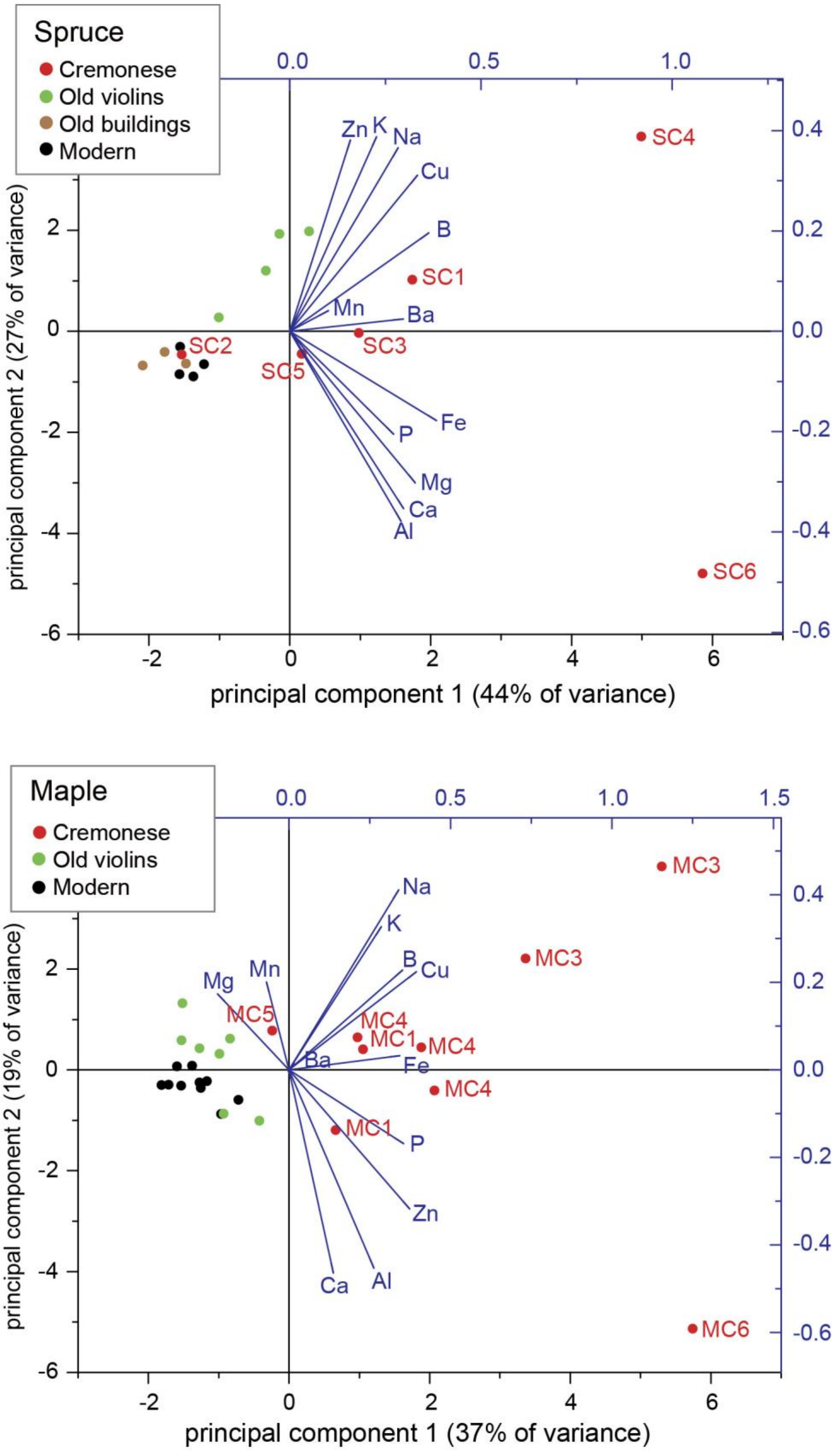

Figure S18. Elemental profiles of Cremonese spruces and maples, represented by principal component analysis biplots.

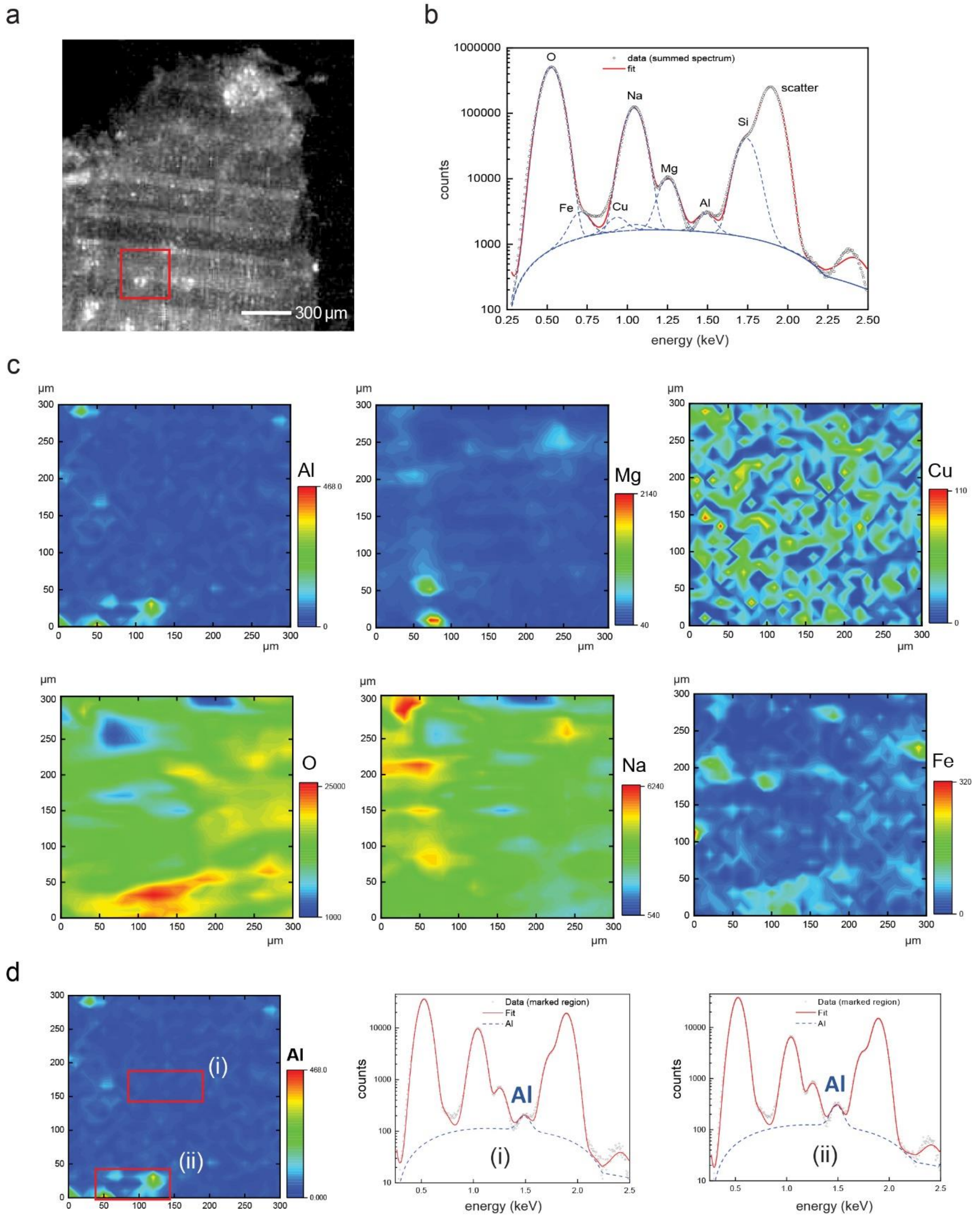

Figure S19. Elemental distributions in Stradivari spruce (SC4). (a) Brightfield microscope image of wood flake. The red box is analyzed for X-ray fluorescence. (b) Cumulative X-ray fluorescence spectra with curve fitting for different elements. (c) Spatial mapping of individual elements in the wood matrix. (d) Curve fitting of Al in low-intensity (i) and high-intensity (ii) areas.

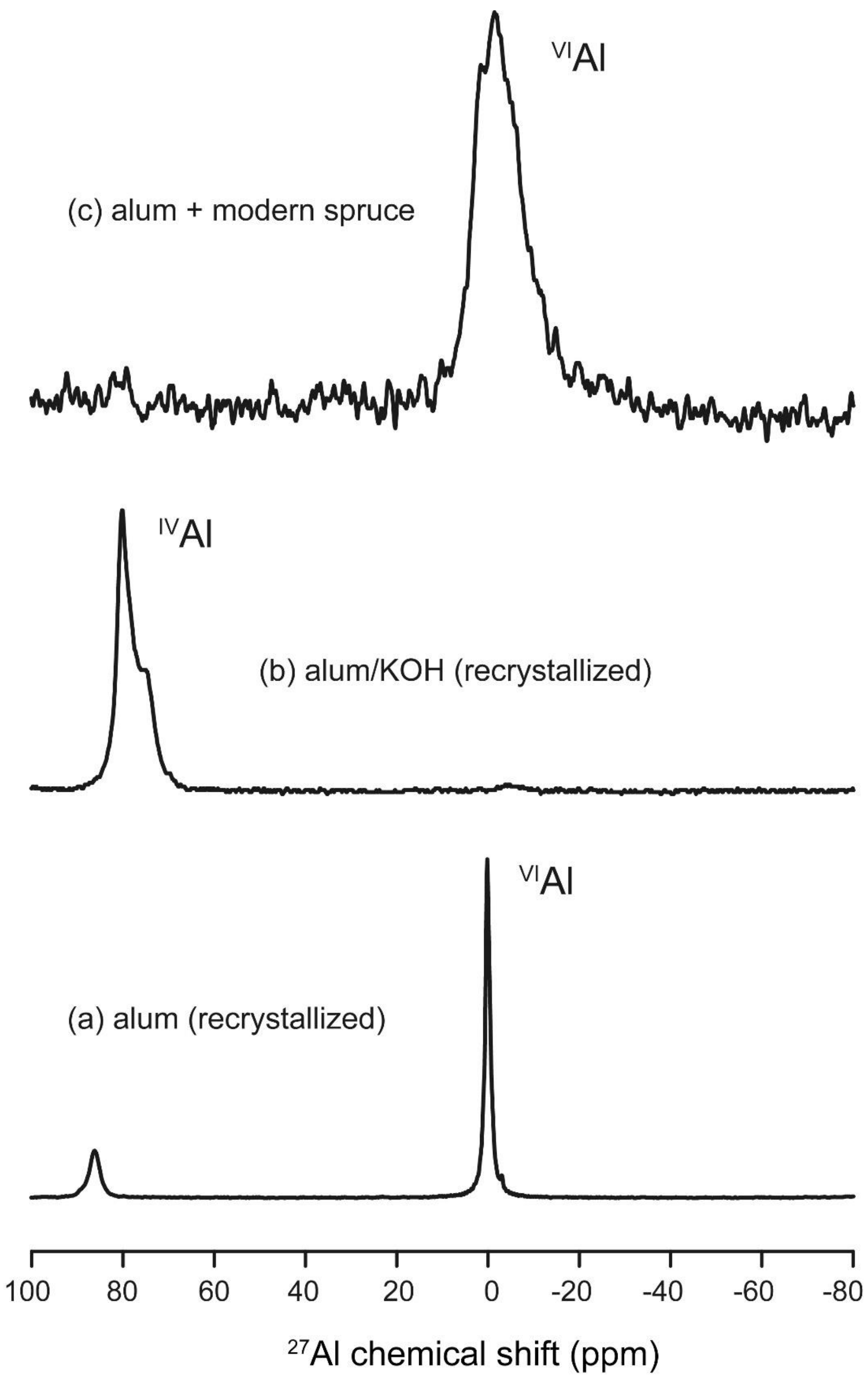

Figure S20. ^{27}Al solid-state NMR spectra of aluminum model compounds: (a) powder obtained from oven drying of alum aqueous solution; (b) powder obtained from oven drying of alum aqueous solution adjusted to pH 14 with KOH; (c) modern spruce (SM5) treated with 1% alum solution and washed three times with deionized water. Chelated Al in the wood shows broader peaks compared to the inorganic crystals.

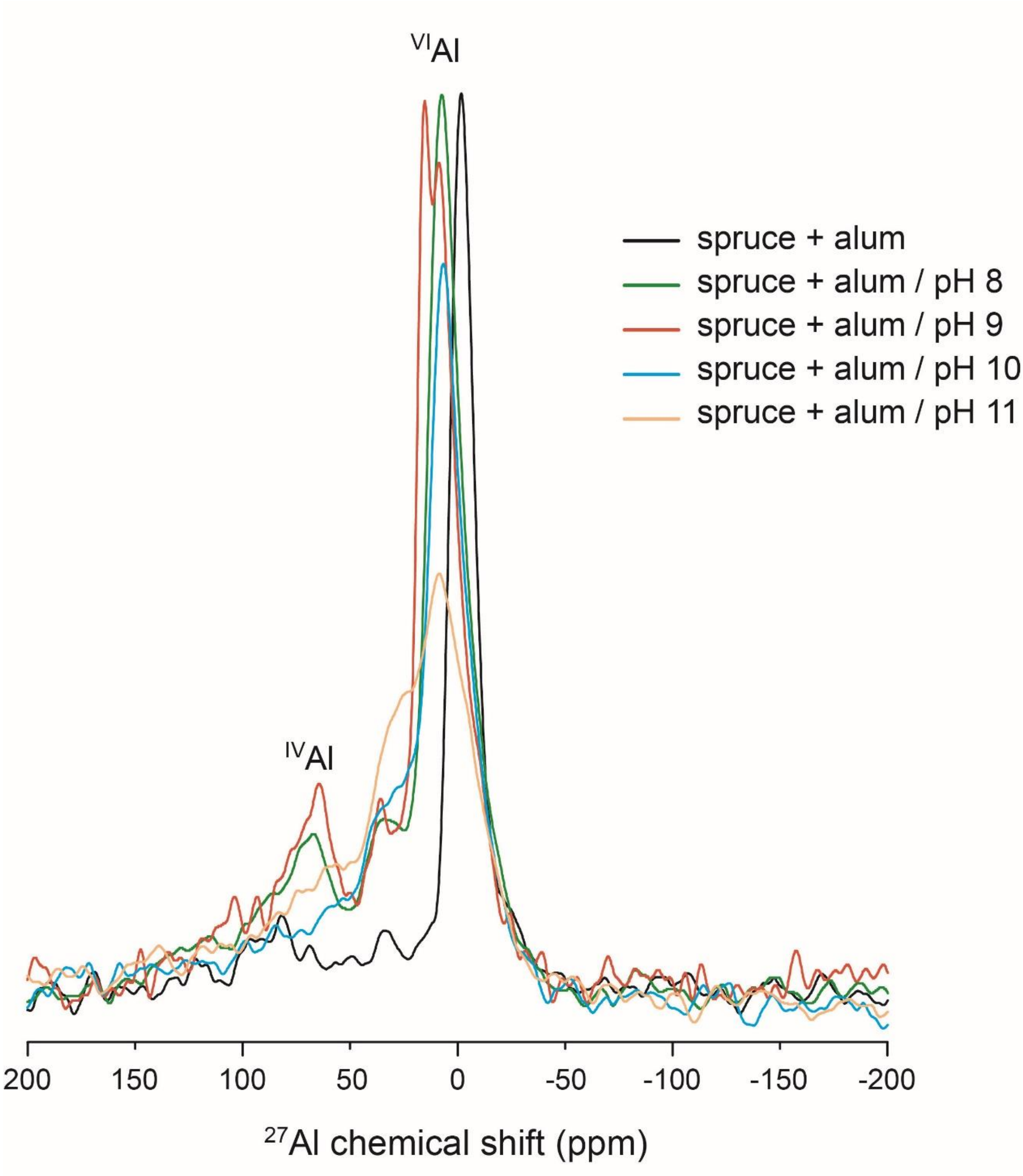

Figure S21. ^{27}Al solid-state NMR spectra of alum/alkaline-treated spruce. Modern spruce (SM5) was immersed in 1% $KAl(SO_4)_2$ (alum) solution for 3 days, followed by immersion into alkaline buffers of different pH values (8, 9, 10, and 11).

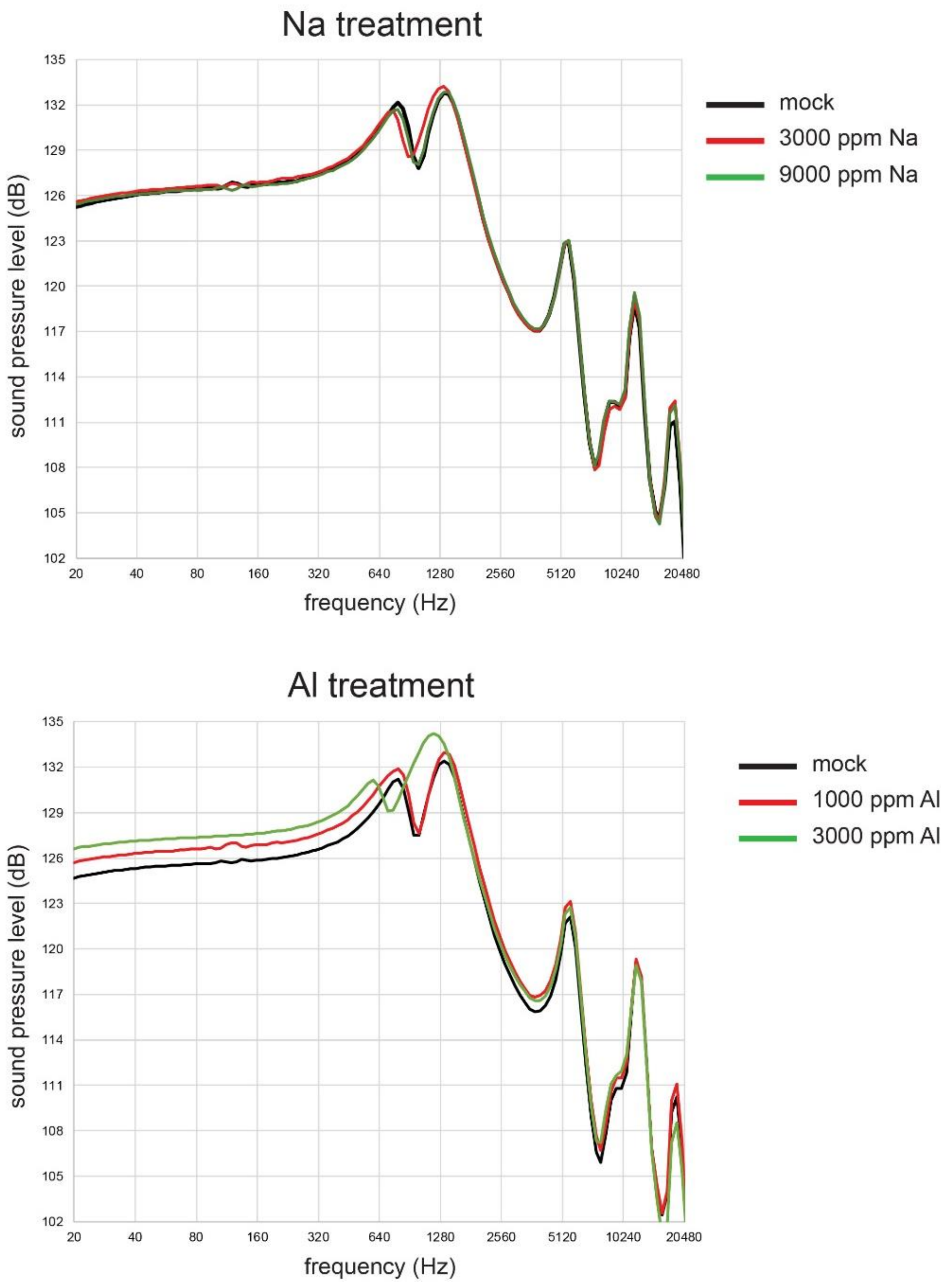

Figure 22. Frequency response curves of cellulosic earphone diaphragms impregnated with Na and Al ions. Mock control is solvent only. The curves are representative of three independent experiments. Adding 1000 and 3000 ppm of Al ion enhances bass frequency response (below 500 Hz) by 1 and 2 dB, respectively.

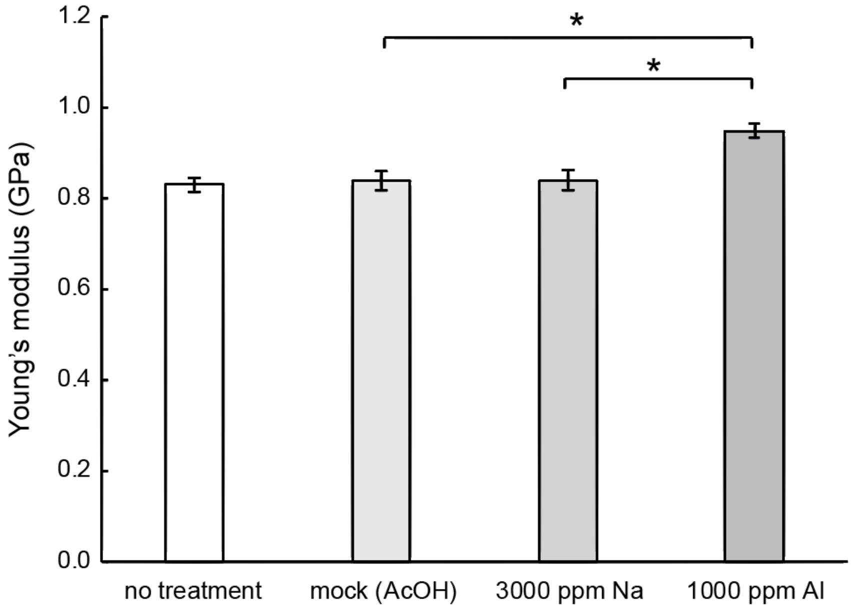

Figure S23. The mechanical effects of aluminum impregnation. Test strips made of cellulosic material are impregnated with Na and Al ions. Mock control is solvent only. The increase in Young's modulus caused by Al addition is statistically significant (n=3, * denotes $p < 0.05$ by two-tailed Welch's t-test).

IV. References

[1] J. Southon, A. Magana, *Radiocarbon* **2010**, *52*, 1371-1379.

[2] M. Stuiver, P. J. Reimer, R. W. Reimer, *CALIB 7.1* Online program: www.calib.org, **2019**.

[3] P. J. Reimer, E. Bard, A. Bayliss, J. W. Beck, P. G. Blackwell, C. B. Ramsey, C. E. Buck, H. Cheng, R. L. Edwards, M. Friedrich, *Radiocarbon* **2013**, *55*, 1869-1887.

[4] B.-J. Lu, J.-R. Li, H.-C. Tai, W. Cai, H.-H. Tseng, Y.-T. Hsieh, *Sci. Rep.* **2019**, *9*, 13253

[5] V. De Andrade, A. Deriy, M. Wojcik, D. Gürsoy, D. Shu, T. Mooney, K. M. Peterson, A. Glowacki, K. Yue, X. Yang, *SPIE* **2016**, *9967*, 99670H.

[6] M. Du, R. Vescovi, R. Chard, N. Kasthuri, C. Jacobsen, E. Dyer, D. Gürsoy, *An automated pipeline for the collection, transfer, and processing of large-scale tomography data*, Biophotonics Congress: Biomedical Optics Congress, **2018**.

[7] R. Chard, K. Chard, J. Alt, D. Y. Parkinson, S. Tuecke, I. Foster, *Ripple: Home automation for research data management*, *IEEE 37th International Conference on Distributed Computing Systems Workshops*, **2017**.

[8] D. Gürsoy, F. De Carlo, X. Xiao, C. Jacobsen, *J. Synchrotron Radiat.* **2014**, *21*, 1188-1193.

[9] B. A. Dowd, G. H. Campbell, R. B. Marr, V. V. Nagarkar, S. V. Tipnis, L. Axe, D. P. Siddons, *SPIE Proceedings* **1999**, *3772*, 224-237.

[10] R. Vescovi, M. Du, V. d. Andrade, W. Scullin, D. a. Gürsoy, C. Jacobsen, *J. Synchrotron Radiat.* **2018**, *25*, 1478-1489.

[11] H. Peng, A. Bria, Z. Zhou, G. Iannello, F. Long, *Nat. Protoc.* **2014**, *9*, 193.

[12] C. Piamonteze, U. Flechsig, S. Rusponi, J. Dreiser, J. Heidler, M. Schmidt, R. Wetter, M. Calvi, T. Schmidt, H. Pruchova, *J. Synchrotron Radiat.* **2012**, *19*, 661-674.

[13] V. Solé, E. Papillon, M. Cotte, P. Walter, J. Susini, *Spectrochim. Acta B* **2007**, *62*, 63-68.

[14] B. Ravel, M. Newville, *J. Synchrotron Radiat.* **2005**, *12*, 537-541.

[15] P. Ildefonse, D. Cabaret, P. Sainctavit, G. Calas, A.-M. Flank, P. Lagarde, *Phys. Chem. Min.* **1998**, *25*, 112-121.

[16] R. L. Johnson, K. Schmidt-Rohr, *J. Magn. Reson.* **2014**, *239*, 44-49.

[17] Y. J. Hsu, C.-C. Chen, C.-H. Huang, C.-H. Yeh, L.-Y. Liu, S.-Y. Chen, *Biomed. Opt. Express* **2017**, *8*, 3005-3016.

[18] S.-Y. Chen, Y. J. Hsu, C.-H. Yeh, S.-W. Chen, C.-H. Chung, *J. Opt.* **2015**, *17*, 035301.

[19] a) S.-Y. Chen, C.-S. Lu, C.-H. Yeh, *Biomed. Opt. Express* **2014**, *5*, 338-347; b) H.-C. Tai, P.-L. Chen, J.-W. Xu, S.-Y. Chen, *Opt. Express* **2020**, *28*, 38831-38841.

[20] S. Schlachter, S. Schwedler, A. Esposito, G. K. Schierle, G. Moggridge, C. Kaminski, *Opt. Express* **2009**, *17*, 22747-22760.

[21] ASTM, *Standard test method for measuring vibration-damping properties of materials (E756)*, ASTM International, West Conshohocken, PA, **2004**.

[22] Ø. Hammer, D. A. Harper, P. D. Ryan, *Palaeontol. Electron.* **2001**, *4*, 1-9.

[23] a) J. Nagyvary, J. A. DiVerdi, N. L. Owen, H. D. Tolley, *Nature* **2006**, *444*, 565; b) J. Nagyvary, R. N. Guillemette, C. H. Spiegelman, *PloS one* **2009**, *4*, e4245.

[24] H. C. Tai, G. C. Li, S. J. Huang, C. R. Jhu, J. H. Chung, B. Y. Wang, C. S. Hsu, B. Brandmair, D. T. Chung, H. M. Chen, J. C. Chan, *Proc. Natl. Acad. Sci. U. S. A.* **2017**, *114*, 27-32.
